# Ultrasonic-assisted ternary deep eutectic solvent extraction of polysaccharides from fermented black beans (Dan-Dou-Chi): structural features, antioxidant, and hypoglycemic activities

**DOI:** 10.1016/j.fochx.2026.104103

**Published:** 2026-06-15

**Authors:** Mengjie Xu, Haobo Ma, Yuan Yuan, Jing Guo, Jiao Kang, Weirong Jie, Yunxi Yang

**Affiliations:** a Department of Biological Sciences, XinZhou Normal University, Xinzhou, Shanxi 034000, China; b Guangdong Provincial Engineering Technology Research Center for Innovative Drugs and Bioproducts, School of Pharmacy, Guangdong Medical University, Dongguan 523808, China; c Shanxi Zhendong Pharmaceutical Co., Ltd. Changzhi, Shanxi 047100, China; d Guangzhou University of Chinese Medicine faculty of Chinese medicine, China

**Keywords:** *Semen sojae praeparatum*, Polysaccharides, Ultrasonic extraction, Ternary deep eutectic solvents, Antioxidant activity, Enzyme inhibition

## Abstract

*Semen Sojae Praeparatum* polysaccharides (SSPP-UD) were extracted using an ultrasonic-assisted ternary deep eutectic solvent (UD; choline chloride: tartaric acid:ethylene glycol, 2:1:1) optimised by response surface methodology (79 min, 35% water, 30 mL/g, 65 °C). This yield reached 115.37 ± 1.1 mg/g, 4.8- and 3.0-fold higher than hot-water and ultrasonic-assisted water extraction, respectively. SSPP-UD contained predominantly low-molecular-weight fractions (1.50 × 10^3^ Da, 55.73%), alongside high-molecular-weight components, with a broad molecular-weight distribution and enrichment in uronic acids, arabinose, and xylose. FT-IR analysis confirmed abundant hydroxyl and carboxyl groups, indicating acidic polysaccharides. SSPP-UD formed stable, negatively charged aqueous aggregates (Dh ∼31.8 μm) and showed antioxidant activity at 3 mg/mL (DPPH, 27.4%; ABTS•^+^, 20.6%; hydroxyl radical, 33.3%), as well as *α*-amylase and *α*-glucosidase inhibition (96.8% and 93.7%; IC_50_, 0.77 and 0.68 mg/mL, respectively). These results demonstrate that ultrasound-assisted ternary DES extraction is an efficient, green strategy for obtaining bioactive polysaccharides from fermented black beans for functional food applications.

## Highlights

• Ternary DES-ultrasound extraction improved SSP polysaccharide yield.
• Optimized extraction yielded 115.37 mg/g SSP polysaccharides.
• SSPP-UD showed low-Mw fractions and enriched uronic acids.
• SSPP-UD exhibited antioxidant and enzyme inhibitory activities.
• Fermentation enhanced polysaccharide extractability and bioactivity.

## 1 Introduction

Natural polysaccharides from functional foods and medicinal plants exhibit diverse bioactivities, including antioxidant, hypoglycemic, and immunomodulatory effects (Benalaya et al., 2024; Chen et al., 2022; Dong et al., 2023). Legume-derived polysaccharides, particularly from fermented products, are promising bioactive compounds, because fermentation can alter molecular weight, branching, and monosaccharide composition, thereby improving solubility, bioavailability, and function (Chen et al., 2024; Lin et al., 2023; Liu et al., 2024; Qiao et al., 2022). For example, Douchi (fermented by *Aspergillus* and *Bacillus* spp.) exhibits strong antioxidant activity (Sui et al., 2024; Wang et al., 2008); natto polysaccharides inhibit glucose absorption (Matsumoto et al., 2020); and cheonggukjang polysaccharides modulate immunity via macrophage activation (Cho et al., 2015; Lee et al., 2013), highlighting fermentation’s role in enhancing polysaccharide bioactivity. However, systematic studies integrating extraction optimisation, structural characterisation, and bioactivity evaluation remain limited.

*Semen Sojae Praeparatum* (SSP), a traditional fermented black bean product produced with auxiliary materials such as mulberry leaves and sweet wormwood, remains an underexplored source of bioactive polysaccharides (Guo et al., 2024; Li et al., 2021; Nuerxiati et al., 2019; Yang et al., 2020). Fermentation can modify molecular weight, branching, and monosaccharide composition, enhancing extractability and functional properties (Lei et al., 2022; Xu et al., 2017; Zhou & Huang, 2022). Nevertheless, comprehensive studies that combine extraction optimisation with physicochemical/structural characterisation and bioactivity assessment of SSP polysaccharides (SSPPs) are lacking.

Conventional extraction methods, such as hot-water extraction (CE), are often inefficient for complex matrices, resulting in low polysaccharide recovery and long processing times (Leong et al., 2021; Md Yusoff & Shafie, 2024). Green extraction approaches, including ultrasonic-assisted extraction (UAE) and deep eutectic solvents (DESs), offer more sustainable and effective alternatives. UAE employs cavitation-induced energy to disrupt plant matrices, improve solvent penetration, and enhance mass transfer, thereby increasing polysaccharide yields (Kumar et al., 2021; Leong et al., 2021). DESs, composed of hydrogen bond donors (HBDs) and acceptors (HBAs), are biodegradable, low-toxicity solvents with strong solvating capacity for polar biomolecules, including polysaccharides (Nian & Li, 2022; Posta et al., 2022).

DESs have been used to extract polysaccharides from diverse sources, typically requiring source-specific formulations. For example, choline chloride/1,6-hexanediol DES has been applied to extract tea polysaccharides from Anji white tea (Xia et al., 2023b); choline chloride/lactic acid DES has been used for algal polysaccharides from *Asparagopsis taxiformis* (Yang et al., 2026); and a ternary DES comprising choline chloride, guaiacol, and lactic acid has been used for fungal polysaccharides from *Ganoderma lucidum* (Li et al., 2024b). These studies highlight the tunability of DES composition for different biomass sources and often report higher yields than conventional methods (Guo et al., 2021a; Xia et al., 2023a). Morais et al. (2020) investigated DES–polysaccharide interactions, showing that combining choline chloride with oxalic acid can enhance product quality and purity, achieving up to 8.6-fold higher efficiency than hot-water extraction (Zhang et al., 2020). Although many DESs are liquid near room temperature, their high viscosity often requires mild heating, stirring, or ultrasound to improve fluidity and extraction efficiency (Smith et al., 2014b). Combining UAE with DESs can further improve recovery of bioactive compounds by coupling improved mass transfer with DES solubilisation, thereby increasing extraction of polyphenols, flavonoids, and polysaccharides (Bertolo et al., 2021; Xu et al., 2025a).

Binary DESs (BDESs) with simple compositions often provide limited selectivity and process control, particularly in matrices where polysaccharides coexist with phenolics or other biomolecules (Feng et al., 2023; Li et al., 2024b). Ternary DESs (TDESs) incorporate a third component (e.g., a secondary hydrogen-bond donor), enabling fine tuning of polarity, viscosity, acidity, and hydrogen-bonding capacity. This tunability improves solvation and strengthens interactions with highly polar macromolecules such as polysaccharides, which contain abundant hydroxyl and carboxyl groups and glycosidic linkages (Sathasivam et al., 2026). Plant polysaccharides are heterogeneous, highly polar macromolecules embedded in complex cell-wall matrices, and their extraction requires solvents that balance solubility, selectivity, and mass transfer (Yahaya et al., 2024). By optimising hydrogen-bond networks, adjusting acidity to loosen matrix structures, and lowering viscosity, TDESs can collectively improve extraction efficiency (Bertolo et al., 2021; Xu et al., 2025a). Consistent evidence indicates that introducing a third component increases yield and solubilisation relative to BDESs. For example, ternary DESs produced higher protein partitioning efficiency (71.9% vs. 21.0%), attributable to improved solute–solvent interactions and mass transfer (Zhang et al., 2016). In polysaccharide extraction, systems such as choline chloride/guaiacol/lactic acid yielded higher recovery and solubilisation of *Ganoderma lucidum* polysaccharides than binary formulations (Li et al., 2024b). Mechanistically, TDESs generate more complex hydrogen-bond networks that increase the number and diversity of interaction sites, while modulating melting point and viscosity, thereby enhancing solubility and selectivity for highly polar macromolecules (Al-Akayleh et al., 2025). However, the use of TDESs for polysaccharide extraction from fermented plant materials remains limited, motivating the present study.

To address these gaps, we adopted a multidimensional strategy with three objectives: (1) compare SSPPs with black bean polysaccharides (BBPs) to define fermentation-induced changes in polysaccharide composition and structure; (2) develop an ultrasonic-assisted TDES (UD) extraction approach to overcome the limitations of binary systems and improve polysaccharide recovery from SSP; and (3) optimise extraction variables using single-factor experiments and response surface methodology (RSM), while characterising TDES properties, polysaccharide structure, antioxidant capacity, and inhibitory activity against *α*-glucosidase and *α*-amylase to probe structure–function relationships.

By combining TDESs with ultrasonic assistance, this study seeks to establish optimal conditions for green, efficient extraction of SSPPs and to comprehensively evaluate their structural features and bioactivity. The integrated optimisation and characterisation provide a basis for developing natural antioxidants and functional ingredients from fermented legumes, supporting advances in green extraction technologies, nutritional pharmacology, and the valorisation of traditional fermented foods.

## 2 Materials and methods

### 2.1 Materials and reagents

*Semen Sojae Praeparatum* (SSP; dried fermented black soybean) and its unfermented precursor, black beans (BB; glycine max seeds), were purchased from Beijing Tongrentang Co., Ltd. (Beijing, China). SSP is a fermented soybean product prepared primarily from black soybeans, with mulberry leaves (*Mori Folium*) and sweet wormwood (*Artemisiae Annuae Herba*) added as auxiliary materials during fermentation, as specified in the Chinese Pharmacopoeia. The BB sample comprised whole, dried seeds with a black seed coat, whereas SSP consisted of dried, whole fermented beans with a wrinkled surface and characteristic aroma. Both materials were authenticated by Professor Jun Yin (Shenyang Pharmaceutical University) and deposited at the Guangdong Provincial Key Laboratory (Dongguan, China) under accession numbers 20250111 TCM-01 (SSP) and 20250111 TCM-02 (BB). All reagents were of analytical grade; sources are provided in Supplementary Material S1.

### 2.2 Synthesis of BDESs and TDESs

Six BDESs were prepared using choline chloride (ChCl) as the hydrogen-bond acceptor (HBA) and polyols as hydrogen-bond donors (HBDs): 1,3-butanediol (1,3-Buta), 1,4-butanediol (1,4-Buta), 1,2-propylene glycol (1,2-PG), 1,3-propylene glycol (1,3-PG), ethylene glycol (EG), and glycerol (Gly). ChCl was chosen for its ability to form stable hydrogen-bond networks with HBDs and is suitable for extracting polar biomacromolecules; the polyols were selected for strong hydrogen-donating capacity and adjustable viscosity, both important for extraction performance (Feng et al., 2023; Qu et al., 2023; Wang et al., 2024a).

Each BDES was prepared by mixing HBA and HBD at a 1:2 molar ratio and stirring at 600 rpm in a water bath at 80 °C until a clear liquid formed (Yue et al., 2012). Based on prior studies, a 1:2 HBA:HBD ratio provides a favourable balance between viscosity and hydrogen-bonding strength, improving solvent fluidity and mass transfer during extraction (Qu et al., 2023). The resulting DESs were stored in sealed containers at room temperature until use.

To further expand solvent tunability, TDESs were prepared by adding an organic acid (D,L-malic acid, MA; oxalic acid, OA; D,L-tartaric acid, TA; citric acid, CA; or D,L-lactic acid, LA) as a secondary HBD to the superior ChCl–EG system. This third component modulates acidity, polarity, and hydrogen bonding—parameters that govern polysaccharide release from plant matrices (Feng et al., 2023).

### 2.3 Physicochemical characteristics of TDESs

The physicochemical properties of the synthesised TDESs (viscosity, density, pH, and hydrogen bonding) were characterised. Viscosity was measured using an SNB-1 viscometer (Shanghai Jitai Electronic Technology Co., Ltd., Shanghai, China) at a predetermined temperature. Density was determined using a DMA4100 densitometer (Anton Paar GmbH, Graz, Austria) at a pre-defined temperature. pH was measured using a PHS-25 pH metre (Shanghai Jiepeng Scientific Instruments Co., Ltd., Shanghai, China). Hydrogen-bond formation between HBD and HBA in DES-9 (choline chloride/tartaric acid/ethylene glycol; molar ratio 2:1:1), one of the 21 DES systems screened, was verified by FT-IR and NMR spectroscopy. FT-IR spectra were collected on a Nicolet IS 50 FTIR spectrometer (Thermo Fisher Scientific, Waltham, MA, United States) over 400–4000 cm^−1^ (Yue et al., 2012). NMR spectra were acquired on a Bruker ASCEND 600 MHz NMR spectrometer equipped with a CryoProbe (Bruker BioSpin GmbH, Germany). Data were processed in Mestrenova (Bruker, Germany), as described by AlNashef et al. (2015).

### 2.4 Computational procedure for DFT calculations

DFT calculations were performed to investigate intermolecular hydrogen-bonding interactions within the DES system, including geometry optimisation, interaction-energy calculations, hydrogen-bond distance analysis, and electrostatic potential (ESP) mapping. Computational details (software, level of theory, basis set, frequency validation, BSSE treatment, etc.) are provided in Supplementary Material S2.

### 2.5 Extraction and recovery of SSPPs

SSPP extraction was performed as previously reported, with minor modifications (Wang & Li, 2022). Samples were ground, passed through a 60-mesh sieve, and freeze-dried to < 5% moisture to improve stability and reproducibility (Monsoor, 2005; Thuy et al., 2020; Xu et al., 2018).

For extraction, 1.0 g of sample was mixed with DES at a liquid-to-solid ratio of 30 mL/g in a 50-mL flask and subjected to ultrasonic-assisted extraction in an ultrasonic bath (KS-300GDV, Kunshan Shumei Ultrasonic Instrument Co., Kunshan, China) at 40 kHz and 300 W. Ultrasonication enhances cell-wall disruption, solvent penetration, and mass transfer, which is advantageous for viscous DESs (Kumar et al., 2021; Leong et al., 2021).

For initial screening of 21 DES systems, standardised conditions were applied: 30% water content, 60 min extraction, 80 °C ultrasonic temperature, and a 30 mL/g liquid-to-solid ratio. Prior studies indicate that temperatures of ∼60–100 °C, extraction times of 40–120 min, and liquid-to-solid ratios of 15:1–35:1 (mL/g) effectively reduce DES viscosity, improve fluidity, and promote solute diffusion (Yu et al., 2024b). Moderate water contents (20–40%) can preserve the hydrogen-bonding network while lowering viscosity, thereby enhancing polysaccharide solubilisation (Dai et al., 2013; Nolasco et al., 2022; Ruesgas-Ramón et al., 2017). Fixing water content at 30% ensured consistent mass transfer and enabled fair comparison across DESs. These settings were used only for preliminary screening; optimal conditions were subsequently established via single-factor experiments and RSM.

To assess extraction performance, two control groups were included: conventional extraction (CE) and ultrasonic-assisted water extraction (UW). For UW, DES was replaced with deionised water; CE was performed in a boiling water bath for 6 h using the same liquid-to-solid ratio. Differences among CE, UW, and ultrasonic-assisted DES extraction (UD) were attributed to methodological differences and optimisation rather than experimental variability. Conditions are summarised in Table S1.

After extraction, suspensions were centrifuged at 8000 rpm for 20 min, and the supernatants were collected. Polysaccharides were precipitated by adding four volumes of absolute ethanol and incubating at 4 °C for 24 h, then collected by centrifugation. The precipitates were deproteinised with 30% trichloroacetic acid (15 mL) in 15 mL water, followed by vortexing for 30 min and incubation at 4 °C for 30 min, and then re-precipitated with four volumes of ethanol at 4 °C for 12 h. The final precipitates were collected and lyophilised.

Polysaccharides obtained by CE, UD, and UW were denoted SSPP-CE, SSPP-UD, and SSPP-UW, respectively. Polysaccharide content was determined using the phenol-sulfuric acid method with D-glucose as the standard (Zavřel et al., 2018), with the calibration curve y = 19.4560X − 0.0208 (R^2^ = 0.9993). Yield was calculated as:

$$\text{Polysaccharide yield}\;\left(\mathrm{mg}/g\right)=\frac{C\times V\times N}{M}\times 100\%$$

where M is the mass of the sample powder (g), N is the dilution factor, V is the volume of the polysaccharide solution (mL), and C is the polysaccharide concentration in the test solution (mg/mL).

### 2.6 Single-factor experiments and response surface methodology (RSM) for process optimisation

DES-9, which provided the highest polysaccharide extraction efficiency, was selected for further optimisation. Single-factor experiments evaluated the effects of four variables on polysaccharide yield: DES water content (10%, 20%, 30%, 40%, and 50%), extraction time (30, 45, 60, 75, and 90 min), ultrasonic temperature (40, 50, 60, 70, and 80 °C), and solvent-to-solid ratio (20, 25, 30, 35, and 40 mL/g).

RSM was used to optimise polysaccharide extraction from SSP. Based on single-factor results, a three-level, four-factor Box–Behnken design (BBD) was constructed (Wang et al., 2024b) (Table S2), comprising 29 runs (Table S3). The polysaccharide yield was designated as the response variable for model fitting. Design-Expert 8.0.6.1 was used to build the model, evaluate factor interactions, and predict optimal extraction conditions. The predicted optimum was validated by triplicate confirmatory experiments. Yields were compared across CE, UW, and both optimised and non-optimised ultrasonic-assisted DES-9 extraction (UD-9). SSPP and BBP were also compared to assess method applicability across sample types.

### 2.7 Structural characteristics

#### 2.7.1 Chemical composition analysis

Total carbohydrate (crude polysaccharide) content was determined by the phenol–sulfuric acid method using D-glucose as the standard, with absorbance measured at 490 nm after reaction with phenol and concentrated sulfuric acid. Total reducing sugar content was quantified by the 3,5-dinitrosalicylic acid (DNS) method, in which reducing sugars react with DNS reagent under heat to form a chromogen measured spectrophotometrically, using D-glucose as the calibration standard. Protein content was quantified by the Bradford assay using bovine serum albumin (BSA) as the standard (Bradford, 1976). Total phenolic content was determined by the Folin–Ciocalteu method using gallic acid as the reference Libbey and Walradt (1968). All assays were performed in triplicate using calibration curves prepared under identical conditions.

#### 2.7.2 Hydrodynamic size and zeta potential analysis

The hydrodynamic diameter (Dh) of polysaccharide aggregates in aqueous suspension was measured using dynamic light scattering (DLS) (Malvern Nano-S90, Malvern Instruments, Worcester, UK) (Geng et al., 2024). Dh reflects solution dispersion and aggregation rather than the size of individual molecules. Zeta potential was measured on the same instrument to assess colloidal stability. Detailed measurement procedures and data are provided in Supplementary Materials S3.

#### 2.7.3 Scanning electron microscopy (SEM) analysis

SEM was used to characterise the microstructure of SSP and BB polysaccharide powders (Khoo et al., 2020). Samples were gold-coated to enhance surface conductivity. Images were acquired at 5 kV at 2000× magnification.

#### 2.7.4 Monosaccharide composition and molecular weight (Mw)

Monosaccharide composition was analysed by ion chromatography (ICS5000+; Thermo Fisher Scientific, Waltham, MA, USA) using a Dionex Carbopac PA10 column (4 × 250 mm), as previously described (Geng et al., 2024). Molecular weight distribution was determined by high-performance gel permeation chromatography (HPGPC) (Thermo U3000) with a refractive index detector (RI-20A) and a BRT 105-103-101 column set (8 × 300 mm). Dextran standards were used to relate retention time to log molecular weight, enabling calculation of weight-average (Mw), number-average (Mn), and peak molecular weights (Mp). Peak number and relative abundance were determined via peak area normalisation. Overall molecular weight parameters were calculated as area-weighted averages across all peaks, and polydispersity (Mw/Mn) was used to describe distribution breadth. Because RI signal intensity depends on solute concentration and its distribution across the elution profile, responses may differ among samples. Detailed procedures are provided in Supplementary Materials S4 and S5.

#### 2.7.5 Fourier transform-infrared (FT-IR) spectroscopy and ultraviolet-visible (UV-Vis) absorption spectroscopy analysis

Functional groups were characterised by FT-IR spectroscopy (Thermo Fisher, USA) (Wang et al., 2014b). UV–Vis absorption spectra were recorded on a Cary 60 spectrophotometer (Agilent, USA) to assess potential impurities (Geng et al., 2024). Details are provided in Supplementary Material S6.

### 2.8 *In vitro* antioxidant activity analysis

In vitro antioxidant activity was evaluated using DPPH radical-scavenging, ABTS•^+^ radical cation-scavenging, hydroxyl radical-scavenging, and ferric reducing antioxidant power (FRAP) assays. DPPH activity was measured using a modified method based on Zhang et al. (2022); ABTS•^+^ followed a modified method by Saravanakumar et al. (2021); hydroxyl radical-scavenging followed Chen and Huang (2019); and FRAP followed González-Centeno et al. (2012) with minor modifications. Ascorbic acid (Vc) was used as the positive control. Polysaccharide concentrations ranged from 0.1 to 3.0 mg/mL for DPPH and ABTS assays and from 0.5 to 3.0 mg/mL for hydroxyl and FRAP assays. Detailed procedures, including calculation formulas, are provided in Supplementary Material S7.

### 2.9 Enzyme inhibition activity assay

Alpha-amylase inhibition was measured using a modified method based on Wang et al. (2018a). Alpha-glucosidase inhibition was assessed using a protocol developed with reference to Wang et al. (2018b). Acarbose served as the positive control. All experiments were performed in triplicate. Detailed methods are provided in Supplementary Materials S8.

### 2.10 Statistical analysis

All results are reported as mean ± SD from triplicate experiments. Statistical analyses were performed in SPSS (version 26.0; IBM Corp., Chicago, IL, USA), and graphs were generated in Origin (2019) (OriginLab Corp., Northampton, MA, USA). Comparisons among samples at the same concentration or condition were performed using one-way ANOVA with Tukey’s post hoc test; *p* < 0.05 was considered significant. For RSM experiments, model and variable significance were evaluated by ANOVA. Statistical significance is selectively annotated in figures, with detailed results provided in the Supplementary Tables.

## 3 Results and discussion

### 3.1 Screening and mechanistic insights into DESs for extraction of SSPP

**Fig. 1:**
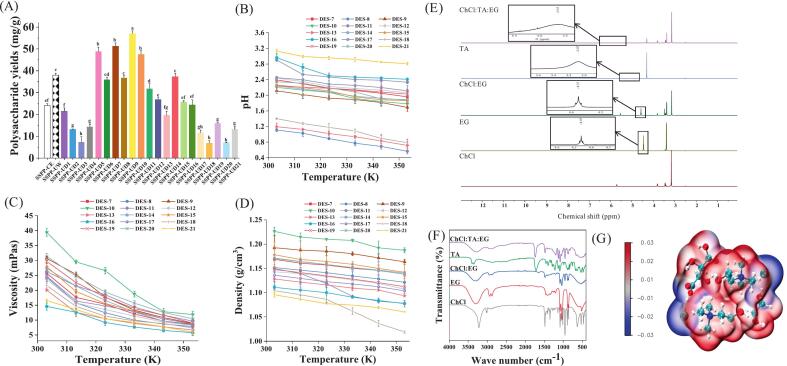
Effect of different deep eutectic solvents (DESs) on the extraction efficiency of *Semen Sojae Praeparatum* polysaccharides (SSPPs) (A) and physicochemical properties of ternary DESs: (B) pH at different temperatures; (C) viscosity at different temperatures; (D) density at different temperatures; (E) ^1^H NMR spectra of choline chloride (ChCl), ethylene glycol (EG), tartaric acid (TA), binary, and ternary DESs; (F) FTIR spectra of ChCl, EG, TA, binary, and ternary DESs; (G) electrostatic potential maps of TDES-9. In panel (A), SSPPs were extracted using three different methods: CE (conventional hot-water extraction, SSPP-CE), UW (ultrasonic-assisted water extraction, SSPP-UW), and UD (ultrasonic-assisted DES extraction, SSPP-UD). Data are presented as mean ± SD (n = 3 independent replicates). Error bars represent the standard deviation. Different lowercase letters indicate statistically significant differences among samples (*p* < 0.05), determined by one-way ANOVA followed by Tukey’s multiple comparison test. Panels (B–D) show physicochemical properties of ternary DESs; statistical comparisons at the same temperature were performed using the same method. Detailed results are provided in the Supplementary Tables.

Polysaccharide extraction from complex matrices is strongly influenced by solvent properties. Six ChCl-based BDESs were synthesised using different HBDs (Table 1) and evaluated at 30% water, which provides moderate hydration that improves fluidity while maintaining the hydrogen-bond network, enabling direct comparisons across systems (Qu et al., 2023). ChCl-EG achieved the highest yield (48.84 ± 2.00 mg/g), exceeding CE (24.07 ± 0.86 mg/g) and UW (37.96 ± 0.94 mg/g), consistent with reports that BDESs enhance polysaccharide solubility via hydrogen bonding (Fig. 1A) (Feng et al., 2023; Qu et al., 2023).

**Table 1:** Compositions and solvent codes for the prepared DESs.

Solvent Code	Combination	Abbreviation	Molar ratio	Water
DES-1	Choline chloride: 1,3-Butanediol	ChCl:1,3-Buta	1:2	30%
DES-2	Choline chloride: 1,4-Butanediol	ChCl:1,4-Buta	1:2	30%
DES-3	Choline chloride: 1,2-Propylene glycol	ChCl:1,2-PG	1:2	30%
DES-4	Choline chloride: 1,3-Propylene glycol	ChCl:1,3-PG	1:2	30%
DES-5	Choline chloride: Ethylene glycol	ChCl:EG	1:2	30%
DES-6	Choline chloride: Glycerol	ChCl:Gly	1:2	30%
DES-7	Choline chloride : Malic acid : Ethylene glycol	ChCl:MA:EG	2:1:1	30%
DES-8	Choline chloride : Oxalic acid : Ethylene glycol	ChCl:OA:EG	2:1:1	30%
DES-9	Choline chloride : Tartaric acid : Ethylene glycol	ChCl:TA:EG	2:1:1	30%
DES-10	Choline chloride : Citric acid : Ethylene glycol	ChCl:CA:EG	2:1:1	30%
DES-11	Choline chloride : Lactic acid : Ethylene glycol	ChCl:LA:EG	2:1:1	30%
DES-12	Choline chloride : Malic acid : Ethylene glycol	ChCl:MA:EG	2:1:2	30%
DES-13	Choline chloride : Oxalic acid : Ethylene glycol	ChCl:OA:EG	2:1:2	30%
DES-14	Choline chloride : Tartaric acid : Ethylene glycol	ChCl:TA:EG	2:1:2	30%
DES-15	Choline chloride : Citric acid : Ethylene glycol	ChCl:CA:EG	2:1:2	30%
DES-16	Choline chloride : Lactic acid : Ethylene glycol	ChCl:LA:EG	2:1:2	30%
DES-17	Choline chloride : Malic acid : Ethylene glycol	ChCl:MA:EG	3:1:2	30%
DES-18	Choline chloride : Oxalic acid : Ethylene glycol	ChCl:OA:EG	3:1:2	30%
DES-19	Choline chloride : Tartaric acid : Ethylene glycol	ChCl:TA:EG	3:1:2	30%
DES-20	Choline chloride : Citric acid : Ethylene glycol	ChCl:CA:EG	3:1:2	30%
DES-21	Choline chloride : Lactic acid : Ethylene glycol	ChCl:LA:EG	3:1:2	30%

To improve control over physicochemical properties, TDESs were prepared by adding a secondary HBD to ChCl-EG at molar ratios of 2:1:1, 2:1:2, or 3:1:2 (Table 1). Their synthesis was confirmed, and pH, viscosity, and density were measured to evaluate their effects on extraction. As shown in Fig. 1B, pH depended on acid identity and the HBA/HBD ratio: oxalic or tartaric acids lowered pH (pH < 1 for DES-8, 13, 18), whereas increasing the EG or ChCl increased pH by reducing the relative acid content and altering hydrogen bonding (Dai et al., 2013; Paiva et al., 2014; Smith et al., 2014a). Polysaccharide yields increased at moderately low pH, likely due to enhanced cell-wall disruption, weakened polysaccharide–protein/phenolic interactions, and reduced viscosity, thus improving ultrasonic cavitation and mass transfer (Imran et al., 2026; Md Yusoff & Shafie, 2025; Shu et al., 2025a; Xia et al., 2023b). In contrast, at pH < 1, extraction efficiency decreased, likely due to acid-catalysed cleavage of glycosidic bonds, generating low-molecular-weight oligosaccharides/monosaccharides, which can be further degraded during ultrasonication by localised high temperature, pressure, and shear (Liu et al., 2018; Qu et al., 2025; Yang et al., 2026). These degradation products are poorly recovered by ethanol precipitation and remain in the supernatant (Shi, 2016; Tai et al., 2020). Together, these findings indicate an optimal pH window that balances solubilisation with structural integrity, consistent with the superior performance of moderately acidic DES-9. As shown in Fig. 1C, viscosity depended on acid type and decreased with increasing temperature. Higher EG fractions lowered viscosity, whereas higher ChCl fractions slightly increased it. Excessive viscosity limits solvent mobility and matrix penetration, reducing polysaccharide release, whereas very low viscosity may weaken hydrogen-bond networks, decreasing selectivity and increasing degradation risk (Qu et al., 2023). As shown in Fig. 1D, TDES densities (1.02–1.23 g/cm^3^) exceeded that of water and decreased with added EG or ChCl and with increasing temperature. Density can influence extraction by affecting solvent–sample affinity and contact; higher density may reduce affinity, whereas lower density may limit contact (Li et al., 2024b; Shahbaz et al., 2012). DES-9 exhibited an intermediate density that may balance these effects.

To elucidate the superior extraction performance of DES-9 (ChCl/TA/EG, 2:1:1), FT-IR, ^1^H NMR, and density functional theory (DFT) calculations were used to examine intermolecular interactions—particularly hydrogen bonding—that govern DES properties and polysaccharide solubilisation (Dai et al., 2013; Paiva et al., 2014; Smith et al., 2014a). In the ^1^H NMR spectra (Fig. 1E), DES-9 exhibited downfield shifts of hydroxyl protons (*δ*_H_ 4.4–5.7 ppm) relative to the individual components and ChCl-EG, indicating hydrogen-bonding interactions among ChCl, TA, and EG. However, because these interpretations rely on selected signals, they should be considered supportive rather than definitive, as comprehensive DES characterisation typically requires multiple complementary methods. FT-IR spectra (Fig. 1F) showed small shifts in the O–H stretching region, further supporting hydrogen-bond interactions among components (Ling, 2020; Zhu et al., 2016). The lack of new absorption peaks suggests no apparent covalent bond formation, suggesting that DES-9 is formed primarily through noncovalent interactions, consistent with established DES formation mechanisms. DFT calculations (Fig. 1G) further indicated that EG can act as a hydrogen-bond bridge between ChCl and TA, generating a flexible interaction network, in agreement with prior theoretical descriptions of DES hydrogen-bond configurations (Aissaoui et al., 2015; Rashid et al., 2023). Collectively, these results support a hydrogen-bonded network in DES-9, which likely underlies its favourable solvent properties and improved polysaccharide extraction.

Among the tested formulations, TDES-9 achieved the highest polysaccharide yield (56.96 ± 1.32 mg/g), exceeding those of BDES (48.84 ± 2.00 mg/g), CE (24.07 ± 0.86 mg/g), and UW (37.96 ± 0.94 mg/g) (Fig. 1A). This improvement likely reflects (1) optimised physicochemical properties via the third component, (2) strengthened hydrogen bonding (supported by spectroscopy), and (3) enhanced compatibility with SSP. Because TDESs have been less explored than binary DESs for polysaccharide extraction, these results extend current knowledge to fermented legume matrices. On the basis of its physicochemical profile and SSPP extraction yield, DES-9 was selected for further optimisation because it consistently outperformed the other formulations for SSPP extraction.

### 3.2 Single-factor optimisation of UD extraction for SSPP

After identifying DES-9 as the optimal DES, single-factor experiments were conducted to refine key extraction parameters. Using a one-variable-at-a-time design, the influence of four parameters on SSPP yield was assessed: DES water content, extraction duration, ultrasonic temperature, and solvent-to-solid ratio (Fig. S1).

#### 3.2.1 Influence of the water content in DES

DESs are structured via hydrogen bonding between a hydrogen-bond acceptor (HBA) and a hydrogen-bond donor (HBD), which governs viscosity, polarity, and solubilisation capacity. In hydrated DESs, water participates in and modifies the hydrogen-bond network, altering solvent properties (Jančíková et al., 2022; Nolasco et al., 2022). In our study (Fig. S1A), polysaccharide yield increased with water content from 10% to 40%, peaking at 40%. At this level, moderate hydration enhanced DES-9 fluidity and mass transfer while preserving sufficient hydrogen-bonded microstructure to support polysaccharide dissolution (Jančíková et al., 2022; Nolasco et al., 2022; Ruesgas-Ramón et al., 2017). At higher water contents, water competes with HBA–HBD interactions and disrupts the network; above ∼40–50%, the eutectic structure can collapse into a DES-in-water regime, lowering solubilisation efficiency despite reduced viscosity (Djikaev & Ruckenstein, 2012; Hammond et al., 2017; Qu et al., 2023). Water effects are DES-specific: hydrophilic DESs often tolerate moderate hydration, whereas excessive water weakens interactions; hydrophobic or multicomponent DESs may be sensitive even at low water content, promoting nanoscale heterogeneity and shifting viscosity, polarity, and solubilisation behavior (Chen et al., 2026; Jani et al., 2021; Ling & Hadinoto, 2022; Malebrán et al., 2025). Accordingly, water content should be optimised and interpreted on a DES-specific basis.

#### 3.2.2 Influence of extraction duration

Extraction duration significantly affects polysaccharide release and degradation during ultrasonication. When extraction time increased from 30 to 90 min (Fig. S1B), yields increased to a maximum at 75 min, consistent with enhanced diffusion and dissolution of polysaccharides into DES-9 under cavitation (Fu et al., 2021). Beyond 75 min, the yields decreased due to the high temperatures and free radical formation during prolonged sonication, which degraded polysaccharides (Haouache et al., 2020).

#### 3.2.3 Influence of solvent-to-solid ratio

The solvent-to-solid ratio is a key determinant of SSPP yield. As shown in Fig. S1C, increasing the ratio from 20:1 to 30:1 increased yield, whereas further increases to 40:1 reduced yield. At ratios below 30:1, solvent volume was insufficient to support effective contact between DES-9 and SSPP, limiting polysaccharide dissolution. A 30:1 ratio provided adequate solvent availability and favourable ultrasound performance, supporting more uniform cavitation and promoting polysaccharide release. At higher ratios (> 30:1), dilution likely weakened DES-9–polysaccharide interactions and reduced extraction efficiency (Peng et al., 2025; Wei & Zhang, 2023; Wu et al., 2025).

#### 3.2.4 Influence of ultrasonic temperature

Temperature critically affects ultrasonic-assisted DES extraction by altering solvent properties and polysaccharide stability (Qu et al., 2023; Wu et al., 2025). The temperature range was selected based on prior ultrasound- and DES-assisted polysaccharide extraction studies, which typically use 40–90 °C: moderate heating lowers viscosity, enhances mass transfer, and improves extraction, whereas excessive heating can promote thermal degradation or structural modification of polysaccharides. Accordingly, a range of 40–80 °C was adopted to capture both the efficiency-enhancing region and the onset of potential degradation effects (Chemat et al., 2017; Dai et al., 2013; Qu et al., 2023). Yield increased from 40 to 70 °C and then slightly decreased at 80 °C (Fig. S1D), reflecting a trade-off between improved mass transfer and temperature-driven degradation. Increasing temperature reduced DES-9 viscosity (Fig. 1C), improved fluidity, promoted matrix penetration, and lowered the cavitation threshold, facilitating ultrasonic-assisted cell disruption and polysaccharide release (Dai et al., 2013; Li et al., 2025c). However, excessive heating can compromise polysaccharide stability, causing partial depolymerisation or structural modification (Cui & Zhu, 2021; Zhang et al., 2015; Zhou et al., 2024). Because 60–80 °C is widely used for polysaccharide extraction, this interval was adopted for response-surface optimisation to balance extraction efficiency with structural preservation (Feng et al., 2023; Qu et al., 2023; Wang et al., 2024d).

### 3.3 Response surface methodology (RSM) optimisation of UD extraction for SSPPs

To refine the extraction process and capture potential parameter interactions, RSM was employed following single-factor experiments. RSM is a powerful statistical tool for optimising multivariable processes and is particularly suited to DES-mediated extraction, where parameter interactions can significantly influence yield (Qu et al., 2023). A four-factor, three-level BBD was constructed (Table S2) using ranges defined by the single-factor results: extraction duration (A: 60–90 min), water content in DES (B: 30–50%), solvent-to-solid ratio (C: 25–35 mL/g), and ultrasonic temperature (D: 60–80 °C). The polysaccharide yield was used as the response (R1). In total, 29 runs were performed, and the results are summarised in Table S3.

Design-Expert software was used to fit a quadratic regression model describing SSPP yield as a function of the extraction parameters. The regression equation was derived as follows:

$$Y=108.07+1.97A-22.04B+1.75C-4.78D+0.10\mathrm{AB}+28.69\mathrm{AC}-37.24\mathrm{AD}-2.72\mathrm{BC}+6.71\mathrm{BD}+14.69\mathrm{CD}-42.38A^{2}-24.87B^{2}-28.05C^{2}-16.99D^{2}$$

where Y represents polysaccharide yield, and *A*, *B*, *C*, *and D* denote the extraction duration, water content in DES, solvent-to-solid ratio, and ultrasonic temperature, respectively. Model significance and adequacy were evaluated by analysis of variance (ANOVA) (Table 2). The model exhibited a high F-value (2208.95, *p* < 0.0001) and R^2^ = 0.9995 (adjusted R^2^ = 0.9991), with a low C.V.% (1.65), indicating that it accounts for nearly all variability in yield and that the experimental data are precise and reproducible.

**Table 2:** Analysis of variance (ANOVA) for the second-order polynomial model.

Source	Sum of squares	Df	Mean square	*F*-value	*p*-value
Model	31847.59	14	2274.83	2208.95	< 0.0001
A-Extraction duration (min)	46.69	1	46.69	45.34	< 0.0001
B-The water content in DES (%)	5828.70	1	5828.7	5659.89	< 0.0001
C-Solvent-to-solid ratio (mL/g)	36.82	1	36.82	35.75	< 0.0001
D-Ultrasonic temperature (°C)	274.37	1	274.37	266.43	< 0.0001
AB	0.04	1	0.04	0.039	0.8466
AC	3293.04	1	3293.04	3197.67	< 0.0001
AD	5547.27	1	5547.27	5386.61	< 0.0001
BC	29.70	1	29.70	28.84	< 0.0001
BD	179.96	1	179.96	174.75	< 0.0001
CD	863.48	1	863.48	838.47	< 0.0001
A^2^	11652.07	1	11652.07	11314.61	< 0.0001
B^2^	4012.32	1	4012.32	3896.12	< 0.0001
C^2^	5102.58	1	5102.58	4954.8	< 0.0001
D^2^	1873.44	1	1873.44	1819.18	< 0.0001
Residual	14.42	14	1.03		
Lack of Fit	13.41	10	1.34	5.30	0.061
Pure Error	1.01	4	0.25		
Cor Total	31862.01	28			
	R^2^ = 0.9995	R^2^ Adj = 0.9991	C.V% = 1.65		

Diagnostic analyses supported model validity. Residuals followed an approximately normal distribution (Fig. S2A), predicted and observed values agreed closely (Fig. S2B), and plots of standardised residuals versus fitted values showed random dispersion around zero (Fig. S2C). Residuals versus experimental run order (Fig. S2D) were also randomly distributed (approximately −3 to +2), indicating no systematic error. Collectively, these results support the high F-value and confirm the predictive performance of the quadratic model.

All linear terms (*A*, *B*, *C*, and *D*), all quadratic terms (*A*^2^, *B*^2^, *C*^2^, and *D*^2^), and most interaction terms (*AC*, *AD*, *BC*, *BD*, and *CD*) were statistically significant (*p* < 0.05), highlighting the inherently nonlinear nature of the extraction process. Water content (*B*) exhibited the strongest effect (F = 5659.89), suggesting that tuning DES physicochemical properties is the dominant driver of extraction efficiency. The significance of all quadratic terms indicates pronounced curvature, supporting the use of RSM. Among interactions, *AB* was not significant (*p* > 0.05), suggesting that extraction duration and water content act largely independently, whereas the remaining interactions were significant, consistent with synergistic effects. Interactions involving extraction duration (*AC* and *AD*) showed particularly high F-values, indicating that time strongly modulates extraction when coupled with solvent availability and temperature. The significant water content–temperature interaction (*BD*) underscores the coupled roles of solvent structure and thermal conditions: moderate levels reduce viscosity, enhance diffusion and cavitation, and improve extraction, whereas excessive water disrupts hydrogen-bonding and elevated temperature induces polysaccharide modification (Cui & Zhu, 2021; Flores García et al., 2025; Imran et al., 2026; Li et al., 2025c; Rente et al., 2022; Tripathy et al., 2026; Xu et al., 2025a). Similarly, the water content-solvent-to-solid ratio interaction (*BC*) reflects a balance between solvent availability and solvation strength; moderate conditions favour extraction, whereas excessive dilution weakens solute–solvent interactions (Feng et al., 2022; Shu et al., 2025b; Xu et al., 2025b).

**Fig. 2:**
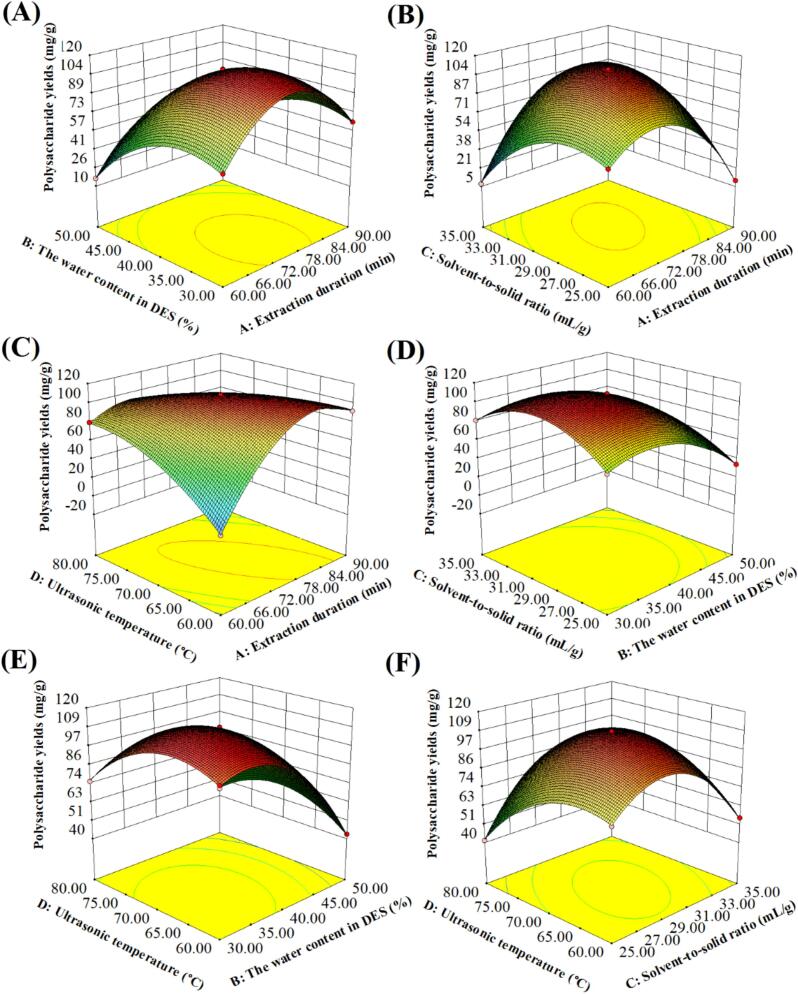
3D response surface plots showing the interactions between extraction variables affecting the yield of the SSPP. (A) Extraction duration and the water content in DES; (B) Extraction duration and solvent-to-solid ratio; (C) Extraction duration and ultrasonic temperature; (D) The water content in DES and solvent-to-solid ratio; (E) The water content in DES and ultrasonic temperature; (F) Solvent-to-solid ratio and ultrasonic temperature.

Three-dimensional response surfaces (Fig. 2) visualise these interactions; the upward convex surfaces indicate that the tested ranges encompass the optimum region. The curvature and contour features demonstrate sensitivity to variable changes and confirm that extraction efficiency is governed by combined factor effects rather than any single parameter (Chen et al., 2019). Overall, polysaccharide extraction is driven by the synergistic interplay between DES physicochemical properties and mass transfer, with water content serving as the primary regulatory factor.

#### 3.3.1 Reproducibility and comparative yield of optimised extraction

**Fig. 3:**
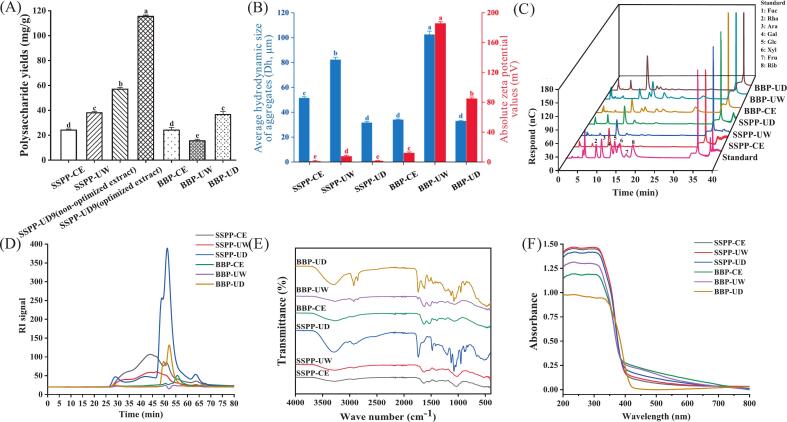
Structural characteristics of SSPPs and BBPs obtained using different extraction methods: (A) polysaccharide yield; (B) Hydrodynamic size and zeta potential; (C) monosaccharide composition; (D) molecular weight distribution; (E) FTIR spectra; (F) UV spectra. Data in panels (A) and (B) are presented as mean ± SD (n = 3 independent replicates). Error bars represent the standard deviation. Different lowercase letters indicate statistically significant differences among samples (*p* < 0.05), determined by one-way ANOVA followed by Tukey’s multiple comparison test.

A BBD within RSM was employed to evaluate the effects of extraction time, DES water content, solvent-to-solid ratio, and ultrasonic temperature on SSPP yield and to determine optimal conditions. The model predicted an optimum at 79 min, 35% DES water content, a 30 mL/g solvent-to-solid ratio, and ultrasonic temperature of 65 °C. Triplicate validation experiments yielded 115.37 ± 1.1 mg/g SSPP, representing a 102.5% increase relative to non-optimised UD extraction (56.96 ± 1.32 mg/g) and substantially exceeding conventional hot-water extraction (CE, 24.07 ± 0.86 mg/g) and ultrasound-assisted water extraction (UW, 37.96 ± 0.94 mg/g). Under the same conditions, BBP-UD yielded 36.53 ± 2.39 mg/g, exceeding CE (24.00 ± 2.28 mg/g) and UW (15.44 ± 1.33 mg/g), while the SSPP yield was ∼3.2-fold higher than BBP, suggesting that fermentation enhances polysaccharide extractability (Fig. 3A). This pattern was reproducible across controlled replicates and is consistent with prior reports that ultrasound-assisted DES enhances extraction via matrix disruption, solvent penetration, and mass transfer, with fermentation-induced structural changes further promoting polysaccharide release (Shu et al., 2025a; Zdanowicz et al., 2018). Because SSP is a fermented product, batch-to-batch variability may arise from differences in raw materials and fermentation conditions; therefore, these results reflect the studied conditions rather than a universal effect.

Pronounced compositional differences were observed across extraction methods (Table 3). For both SSPP and BBP, total carbohydrate content increased from CE to UW to UD, with SSPP-UD highest (76.25%), whereas protein and phenolic contents decreased and were lowest in UD fractions (SSPP-UD protein 3.74%, BBP-UD 2.11%). Reducing sugar content was higher in UD fractions (7.42% for SSPP-UD and 7.15% for BBP-UD), indicating partial glycosidic cleavage. Overall, UD extraction increased yield while enriching carbohydrate-rich fractions and reducing co-extraction of proteins and phenolics relative to CE and UW (Wu et al., 2020; Zdanowicz et al., 2018).

**Table 3:** Chemical composition and purity of polysaccharides extracted from SSP and BB.

Parameters	SSPP-CE	SSPP-UW	SSPP-UD	BBP-CE	BBP-UW	BBP-UD
Total polysaccharide content (%)	57.84 ± 0.55^c^	74.37 ± 1.40^a^	76.25 ± 0.88^a^	59.61 ± 0.55^c^	71.16 ± 1.36^b^	73.84 ± 1.12^ab^
Total reducing sugar content (%)	3.50 ± 0.22^c^	6.00 ± 0.38^b^	7.42 ± 0.63^a^	3.50 ± 0.11^c^	6.50 ± 0.74^b^	7.15 ± 0.59^ab^
Protein content (%)	13.51 ± 0.57^b^	12.79 ± 1.37^b^	3.74 ± 0.28^d^	22.17 ± 1.31^a^	9.55 ± 0.14^c^	2.11 ± 0.51^d^
Total phenolic content (%)	2.24 ± 0.71^b^	1.25 ± 0.42^bc^	0.27 ± 0.06^c^	5.45 ± 0.91^a^	1.45 ± 0.22^bc^	0.10 ± 0.01^c^

*Values are expressed as mean ± SD (n = 3). Different superscript letters within the same row indicate significant differences (*p* < 0.05) according to one-way ANOVA followed by Tukey’s multiple comparison test.

The superior performance of UD extraction likely reflects several concurrent mechanisms (Chemat et al., 2017; Dai et al., 2013). Ultrasonic cavitation disrupts cell walls and facilitates solvent penetration, accelerating polysaccharide release (Vinatoru, 2001). In parallel, the TDES hydrogen-bonding network can interact with polysaccharide hydroxyl and carboxyl groups, weakening associations with proteins and phenolics and thereby promoting dissolution. The mildly acidic environment induces limited glycosidic cleavage, yielding smaller, more soluble fragments. Fermentation further increases polysaccharide accessibility by partially degrading the matrix and reducing intermolecular cross-linking (Liu et al., 2024; Yang et al., 2024). Collectively, these effects support enhanced mass transfer, selective solubilisation, partial depolymerisation, and fermentation-driven matrix modification.

### 3.4 Structural and physicochemical characterisation of polysaccharides: effects of extraction methodology and fermentation

To determine how extraction method and fermentation influence polysaccharide structure and functional potential, SSPP and BBP were comprehensively characterised. The extracts were prepared using CE, UW, and optimised ultrasonic-assisted TDES (UD) extraction. Structural differences associated with extraction strategy were assessed, and their potential implications for bioactivity are discussed below.

#### 3.4.1 Hydrodynamic size and zeta potential analysis

Dynamic light scattering (DLS) was used to determine the Dh of polysaccharide aggregates in aqueous suspension, which reflects dispersion behavior rather than individual polymer chain size. Smaller Dh values indicate improved dispersion and greater surface accessibility, which may translate to higher bioactivity; conversely, larger aggregates can reduce solubility and limit functional interactions (Geng et al., 2024; Yu et al., 2024a). As shown in Fig. 3B, UD-extracted polysaccharides formed the smallest aggregates (SSPP-UD: 31.80 μm; BBP-UD: 33.07 μm), UW-extracted samples formed the largest (SSPP-UW: 82.40 μm; BBP-UW: 102.63 μm), and CE samples were intermediate (SSPP-CE: 51.57 μm; BBP-CE: 34.23 μm), indicating that UD limits aggregation and may enhance solubility and bioactivity.

Zeta potential differed significantly by extraction method, reflecting changes in surface charge and colloidal stability. Larger absolute zeta potentials generally indicate stronger electrostatic repulsion and improved dispersion (Ye et al., 2025). As shown in Fig. 3B, BBP-UW and SSPP-UW exhibited relatively high absolute zeta potentials, consistent with greater stability, whereas BBP-CE and SSPP-CE showed the lowest values, suggesting greater aggregation tendency. UD-extracted samples exhibited intermediate zeta potentials, suggesting moderate stability potentially attributable to extraction-induced structural changes. Ultrasound-assisted extraction can disrupt the cell-wall matrix, reduce hydrodynamic size, and alter physicochemical properties by exposing functional groups (e.g., hydroxyl and carboxyl moieties), thereby modulating intermolecular interactions (Li et al., 2025a; Wang et al., 2023b; Ye et al., 2025).

From a functional perspective, moderate dispersion and surface charge may be advantageous: extensive aggregation limits accessibility, whereas smaller, appropriately charged aggregates can facilitate interactions with free radicals and digestive enzymes. Consistently, polysaccharides with smaller hydrodynamic aggregates, higher absolute zeta potentials, and enriched functional groups have been associated with stronger antioxidant and hypoglycemic activities (Ye et al., 2025).

#### 3.4.2 Microstructure of polysaccharide powders

SEM was used to assess the microstructures of polysaccharide powders from SSP and BB obtained by CE, UW, and UD. Representative images at 2000× magnification are provided in Fig. S4.

In SSPP, CE extracts formed compact, block-like aggregates with smooth surfaces and sharp edges, suggesting minimal structural disruption during conventional extraction. UW extracts showed slightly rougher surfaces and minor fissures, consistent with partial dispersion induced by ultrasonic treatment. Conversely, UD-extracted SSPP (SSPP-UD) showed markedly more fragmented and irregular structures with heterogeneous particle sizes, corrugations, voids, and uneven edges, indicating greater dispersion and microstructural heterogeneity. Among BBP samples, BBP-UD exhibited the most heterogeneous and fragmented morphology, with pronounced surface roughness, irregular fragments, and prominent voids relative to BBP-CE and BBP-UW.

These SEM observations provide qualitative insights into the effects of extraction methods on polysaccharide microstructure; however, SEM alone cannot resolve chemical composition or bioactivity. The trends observed here are consistent with hydrodynamic size and zeta potential measurements, which indicate improved dispersion and reduced aggregation of UD-extracted polysaccharides. Together with compositional and functional analyses, these results help clarify how extraction conditions shape polysaccharide characteristics.

#### 3.4.3 Monosaccharide composition and molecular weight

Monosaccharide composition influences polysaccharide structure and function. Both SSPP and BBP contained fucose, rhamnose, arabinose, galactose, glucose, xylose, fructose, and ribose (Table 4, Fig. 3C), reflecting their shared botanical origin. However, the relative abundances of these two major monosaccharides varied with the extraction method: in SSPP-CE and SSPP-UW, arabinose was present at a higher molar ratio than fructose, whereas in BBP-CE and BBP-UW, fructose exceeded arabinose in molar ratio, although both arabinose and fructose remained the predominant monosaccharides. UD extraction accentuated these differences, with arabinose predominating, fructose markedly reduced, and xylose—undetected in BBP-CE/UW—becoming markedly increased in BBP-UD. These observations indicate that fermentation and extraction jointly shape monosaccharide distribution and may thereby influence polysaccharide functionality.

**Table 4:** Overall molecular weight parameters, molecular weight distribution, and monosaccharide composition of polysaccharides extracted from SSP and BB using different extraction methods.

	SSPP-CE	SSPP-UW	SSPP-UD	BBP-CE	BBP-UW	BBP-UD
Overall molecular weight parameters						
Mw (Da)	1.73 × 10^4^	2.51 × 10^4^	7.54 × 10^4^	7.70 × 10^4^	2.36 × 10^5^	1.08 × 10^5^
Mn (Da)	1.69 × 10^4^	2.45 × 10^4^	7.40 × 10^4^	7.51 × 10^4^	2.30 × 10^5^	1.06 × 10^5^
Mw/Mn	1.02	1.02	1.02	1.03	1.03	1.02

Molecular weight Mw (Da)						
Peak 1	6.90 × 10^4^ (17.86%)	1.04 × 10^5^ (19.37%)	3.98 × 10^5^ (14.77%)	4.29 × 10^5^ (17.59%)	4.75 × 10^5^ (49.17%)	6.12 × 10^5^ (14.24%)
Peak 2	5.75 × 10^3^ (59.18%)	5.51 × 10^3^ (52.96%)	1.05 × 10^3^ (29.50%)	2.52 × 10^3^ (56.68%)	9.45 × 10^3^ (13.00%)	1.72 × 10^4^ (7.07%)
Peak 3	3.73 × 10^3^ (22.96%)	2.85 × 10^3^ (27.67%)	1.50 × 10^3^ (55.73%)	1.55 × 10^3^ (25.73%)	2.18 × 10^3^ (13.26%)	1.89 × 10^3^ (78.69%)
Peak 4	-	-	-	-	1.56 × 10^3^ (24.56%)	-

Monosaccharides compostion (mol%)						
Fucose	5.73	4.2	17.5	2.16	2.54	12.47
Rhamnose	0.56	0.67	1.04	0.9	0.56	1.33
Arabinose	55.48	52.08	50.5	24.4	11.81	60.13
Galactose	7.91	8.86	13.8	8.37	8.96	8.29
Glucose	1.15	2.12	3.04	9.07	19.88	4.93
Xylose	0.56	0.4	2.89	0	0	1.82
Fructose	24.22	26.55	3.53	42.87	51.79	8.67
Ribose	4.38	5.13	7.7	12.23	4.46	2.36

Mw distribution reflects the degree of polymerisation and affects physicochemical properties and bioactivity. High-Mw fractions increase viscosity and can limit solubility and diffusion (Mao et al., 2018), whereas low-Mw fractions often exhibit higher solubility and enhanced biological activity (Lee et al., 2024; Zou et al., 2023). As shown in Fig. 3D and Table 4, SSPPs and BBPs exhibited extraction-dependent Mw profiles. SSPP-CE and SSPP-UW were dominated by intermediate-Mw fractions (∼5.75 × 10^3^ and 5.51 × 10^3^ Da), whereas SSPP-UD showed a larger low-Mw fraction (1.50 × 10^3^ Da, 55.73%) and a broadened overall Mw (7.54 × 10^4^ Da). Similarly, BBP-UD was enriched in low-Mw fractions (1.89 × 10^3^ Da, 78.69%), while BBP-UW retained more high-Mw material (4.75 × 10^5^ Da, 49.17%). These results indicate that both the dominant fractions and the overall Mw distribution are strongly influenced by extraction method.

Mw and Dh describe distinct properties: Mw reflects polymer mass, whereas Dh represents the apparent size of molecules or aggregates in solution and is governed by conformation, solubility, and intermolecular interactions (Morris et al., 2014; Rodriguez-Loya et al., 2023). Accordingly, a higher Mw does not necessarily translate into larger Dh because aggregation can dominate hydrodynamic behavior. In addition, differences in HPGPC signal intensity primarily reflect solute distribution rather than absolute polysaccharide content; therefore, weaker signals in BBP-UW and BBP-UD do not imply lower polysaccharide levels. Notably, SSPP-UD and BBP-UD exhibited similar chromatographic profiles, suggesting convergence of Mw-distribution features under the same extraction system.

The observed changes in monosaccharide composition and Mw likely arise from combined effects of fermentation and extraction. Fermentation can reshape monosaccharide profiles via microbial metabolism and enzymatic activity (Liu et al., 2024; Roullier-Gall et al., 2022). Ultrasonic treatment promotes cell wall disruption and mass transfer, enhancing release of hemicellulose-derived sugars such as arabinose and xylose (Khaire et al., 2021). The mildly acidic, hydrogen-bond-rich DES partially hydrolyses polysaccharide chains and modifies structural organisation (Sun et al., 2022), while ultrasonic cavitation promotes controlled glycosidic cleavage without complete degradation (Cui & Zhu, 2021; Du et al., 2022; Duan et al., 2025; Hu et al., 2026; Xiao et al., 2022; Zhou et al., 2024).

The lower Mw of UD-extracted polysaccharides likely reflects preferential solubilisation of more soluble fractions, moderate structural modification, and partial chain scission rather than extensive degradation. Consistent with prior studies, ultrasound-assisted extraction can modify monosaccharide profiles and molecular structure and may enhance functional properties, including antioxidant and hypoglycemic activities (Xiao et al., 2022; Zheng & Zhang, 2025). These findings support the view that polysaccharide bioactivity is strongly associated with Mw, monosaccharide composition, and functional groups (Zhao et al., 2023a).

#### 3.4.4 FT-IR and UV Spectroscopy

FT-IR spectroscopy was used to identify major functional groups and assess structural features of the extracted polysaccharides (Fig. 3E) (Hong et al., 2021; Jiang et al., 2021). All samples showed characteristic polysaccharide bands, including broad O–H stretching (3600–3200 cm^−1^), C–H stretching (2960–2850 cm^−1^), and strong absorption in the carbohydrate region (1250–950 cm^−1^) attributable to C–O, C–O–C, and glycosidic vibrations (Hong et al., 2021; Synytsya & Novak, 2014). Bands near 883 cm^−1^ indicate *β*-type glycosidic linkages, whereas signals at 1600–1400 cm^−1^ correspond to carbonyl/carboxyl groups in acidic polysaccharides (Shi et al., 2024; Wu et al., 2022).

The SSPP-CE/BBP-CE and SSPP-UW/BBP-UW spectra were highly similar, indicating broadly comparable functional groups (Hong et al., 2021). Although absorption in the glycosidic region (1200–950 cm^−1^) was detected in all samples, the weaker signals in the CE and UW fractions preclude firm conclusions regarding backbone modification. In contrast, the UD extracts (SSPP-UD and BBP-UD) exhibited more evident spectral differences. The stronger absorption at 1250–950 cm^−1^ indicates enhanced carbohydrate-related vibrations, likely reflecting improved extraction efficiency, increased solubilisation, or changes in the local chemical environment rather than polysaccharide backbone cleavage (Pan et al., 2022; Xia et al., 2023a). New peaks at 1735.99 cm^−1^ (SSPP-UD) and 1738.95 cm^−1^ (BBP-UD) correspond to C=O stretching of esterified or protonated carboxyl groups, indicative of uronic acid exposure (Shi et al., 2024; Wu et al., 2022). Overall, the FT-IR data indicate that ultrasonic-assisted TDES extraction primarily alters the intensity and detectability of functional-group signals, reflecting changes in selectivity, solubilisation, and molecular organisation rather than backbone cleavage (Pan et al., 2022). This interpretation is consistent with reports that ultrasound-assisted DES enhances mass transfer and solubilisation, modifying molecular characteristics without disrupting the polysaccharide framework (Shi et al., 2024; Xia et al., 2023a). These structural features—particularly the enhanced glycosidic-region signals and uronic acid-related carbonyl peaks—may underlie differences in biological activity, consistent with the established relationship between polysaccharide structure and bioactivity (Yang et al., 2022).

Building on prior findings on protein content across polysaccharide fractions (Li et al., 2025b), UV spectroscopy was used to further evaluate protein-related features (Fig. 3F). All samples showed an absorption peak near 280 nm, indicating the presence of bound proteins (Mohammed et al., 2020). However, because protein contents among fractions varied significantly (e.g., BBP-CE exhibited a relatively high protein content, whereas SSPP-UD and BBP-UD had lower levels; Table 3), peak intensities were not directly comparable. These variations in protein content likely contribute to the observed spectral differences and may partly influence the physicochemical properties of the extracted polysaccharides.

### 3.5 Analysis of antioxidant properties

**Fig. 4:**
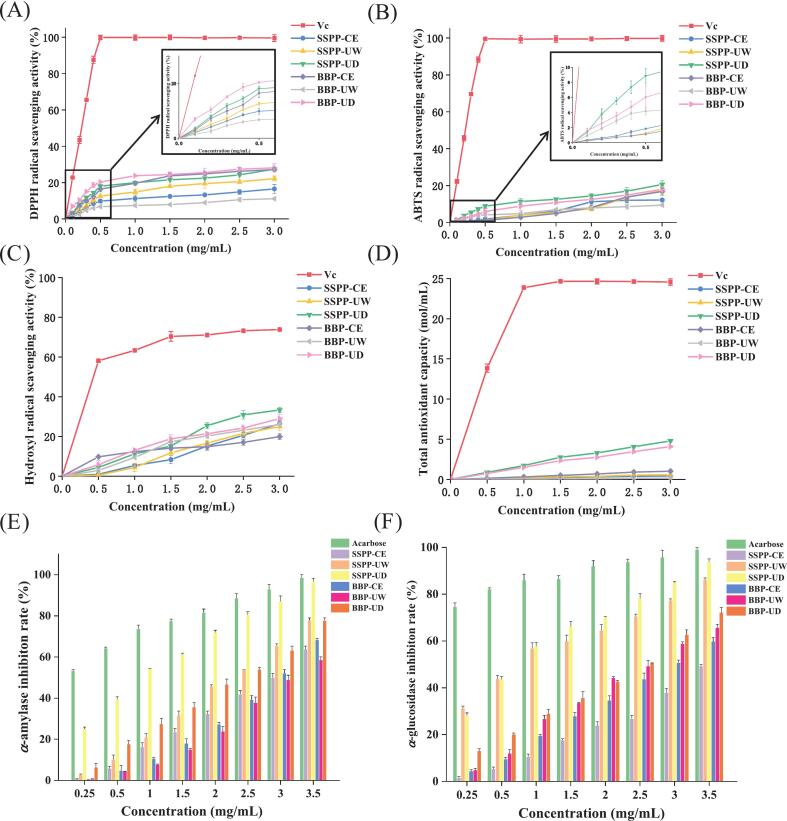
Antioxidant and hypoglycemic activities of SSPPs and BBPs obtained using different extraction methods: (A) DPPH radical-scavenging activity; (B) ABTS•^+^ radical cation-scavenging activity; (C) hydroxyl radical-scavenging activity; (D) total antioxidant capacity; (E) *α*-amylase inhibition; (F) *α*-glucosidase inhibition. Vc represents vitamin C (ascorbic acid), used as the positive control in the antioxidant assays. The concentration ranges were 0.1-3.0 mg/mL for DPPH and ABTS assays, and 0.5-3.0 mg/mL for hydroxyl radical scavenging, T-AOC, and enzyme inhibition assays. Data are presented as mean ± SD (n = 3 independent extraction replicates). Error bars represent the standard deviation. Detailed statistical comparisons among samples at the same concentration (*p* < 0.05), determined by one-way ANOVA followed by Tukey’s multiple comparison test, are provided in the Supplementary Tables.

Oxidative stress promotes the accumulation of damaged macromolecules and contributes to cardiovascular, inflammatory, neoplastic, and metabolic disorders (Chen et al., 2019; Li et al., 2017). The antioxidant activities of SSPP and BBP were evaluated using DPPH, ABTS, and hydroxyl radical (·OH) scavenging assays and total antioxidant capacity (T-AOC) measurements (Fig. 4A-D) over defined concentration ranges (0.1–3.0 mg/mL for DPPH/ABTS; 0.5–3.0 mg/mL for ·OH/T-AOC), with vitamin C as the positive control. All samples showed antioxidant activity, although the magnitude and dose–response profiles varied by assay and extraction method. In the DPPH and ABTS assays, activity increased rapidly at low concentrations and plateaued above 0.5 mg/mL, consistent with saturation rather than linear behavior.

At 3.0 mg/mL, UD-extracted fractions (SSPP-UD and BBP-UD) exhibited higher scavenging activity than the CE and UW fractions (DPPH: 27.4% and 28.1%; ABTS: 20.6% and 18.0%), reflecting a lower molecular weight and altered monosaccharide composition that increase exposure of reactive groups and facilitate radical interactions (Mirzadeh et al., 2020; Qu et al., 2023; Xia et al., 2023a). BBP-UD showed slightly higher DPPH activity, whereas SSPP-UD performed better in ABTS, consistent with assay-specific sensitivity: DPPH primarily reflects hydrogen-donating ability and steric accessibility, whereas ABTS is more responsive to electron transfer in hydrophilic environments (Huang et al., 2005; Schaich et al., 2015). Differences in molecular weight distribution, monosaccharide composition, and uronic acid content may further shape these patterns, because lower molecular weight improves accessibility of reactive groups, sugar composition affects conformation and reactivity, and uronic acids can enhance metal chelation and electron/hydrogen donation (Ji et al., 2022; Wang et al., 2015; Zhou et al., 2025).

In ·OH scavenging assays, SSPP-UD and BBP-UD exhibited the highest activity at 3.0 mg/mL (33.3% and 29.0%, respectively), consistent with the DPPH and ABTS results. In contrast, BBP-CE exhibited relatively higher hydroxyl radical-scavenging activity at lower concentrations. This pattern likely reflects the compositional characteristics of the crude extract rather than the polysaccharide fraction alone. Specifically, BBP-CE contains higher levels of co-extracted proteins and phenolic compounds, which exhibit strong radical-scavenging and metal-chelating activities (Liu et al., 2025; Shahidi & Athiyappan, 2025). At lower concentrations, these small molecules may contribute disproportionately because of their higher reactivity and accessibility in the Fenton-type reaction system used to generate hydroxyl radicals (Fernandes & Coimbra, 2023). Fermentation may also degrade or transform these non-polysaccharide constituents, altering their abundance and contribution to antioxidant activity. At higher concentrations, the contribution of polysaccharides—particularly those with improved structural features in fermented samples—likely becomes dominant, consistent with the overall superior performance of SSPP (Wang et al., 2015). A similar trend was observed in the T-AOC assay (Fig. 4D), in which UD-extracted polysaccharides showed higher reducing capacity at 3.0 mg/mL, likely due to extraction-induced changes in functional groups (e.g., uronic acid residues) and pyranose structures (Shen et al., 2014; Yu et al., 2024a).

The observed antioxidant activities align with reports on plant- and food-derived polysaccharides, for shich *in vitro* efficacy depends on molecular structure and extraction conditions. DPPH and ABTS scavenging typically increases rapidly at low concentrations and then plateaus, whereas ·OH scavenging and reducing power are more strongly structure-dependent. Ultrasound-assisted and DES-based extraction can enhance activity, although effects are system-specific (Ji et al., 2022; Wang et al., 2015; Xia et al., 2023a). UD extraction produced polysaccharides with lower Mw, altered monosaccharide profiles (including arabinose, xylose, and uronic acids), improved dispersion (smaller Dh and appropriate zeta potential), and enriched functional groups, collectively enhancing radical accessibility and bioactivity. Fermentation further depolymerised polysaccharides and exposed reactive sites, whereas ultrasonic-assisted TDES extraction enhanced mass transfer and selective solubilisation (Chen et al., 2005; Qu et al., 2023; Xia et al., 2023a). Thus, the higher antioxidant activity of UD fractions likely arises from the synergistic effects of Mw, monosaccharide composition, functional-group exposure, and dispersion rather than a single determinant.

### 3.6 Analysis of *α*-amylase and *α*-glucosidase inhibitory capacities

*α*-Amylase and *α*-glucosidase mediate starch digestion by hydrolysing starch to oligosaccharides and ultimately glucose. Inhibiting these enzymes reduces glucose release, mitigating postprandial hyperglycemia and potentially slowing diabetes progression.

At 3.5 mg/mL, *α*-amylase inhibition by SSPP-CE, SSPP-UW, SSPP-UD, BBP-CE, BBP-UW, and BBP-UD was 63.89%, 77.84%, 96.75%, 68.29%, 58.68%, and 77.68%, respectively, whereas *α*-glucosidase inhibition reached 49.24%, 86.19%, 93.73%, 59.84%, 65.74%, and 72.18% (Fig. 4E–F). IC_50_ values (0.25–3.5 mg/mL; Table S7) confirmed SSPP-UD as the most potent inhibitor (*α*-amylase, 0.77 mg/mL; *α*-glucosidase, 0.68 mg/mL), with 2–4-fold greater potency than the other fractions. In contrast, BBP samples showed higher IC_50_ values (*α*-amylase, 2.02–3.07 mg/mL; *α*-glucosidase, 2.13–2.90 mg/mL), and SSPP-CE did not reach 50% *α*-glucosidase inhibition (IC_50_ > 3.5 mg/mL). Overall, enzyme inhibition depended strongly on extraction method, with UD-derived polysaccharides consistently exhibiting higher potency. By comparison, crude legume extracts (kidney bean, lentil, chickpea) show weak *α*-amylase and *α*-glucosidase inhibition (IC_50_ ≈ 43–77 and 57–139 mg/mL), whereas a cereal control (bulgur) shows moderate activity (*α*-amylase ≈ 63 mg/mL; *α*-glucosidase ≈ 57.7 mg/mL) (Bayrak, 2026). These comparisons indicate that UD-derived polysaccharide-rich fractions have substantially higher inhibitory potential than typical crude extracts.

The enhanced activity of SSPP-UD likely reflects synergistic effects of fermentation and extraction. Fermentation can remodel polysaccharide structures via enzymatic hydrolysis, reducing molecular weight and altering monosaccharide composition by increasing low-Mw fractions and the relative abundance of arabinose, xylose, and uronic acids (Guo et al., 2021b; Su et al., 2023; Wang et al., 2023a; Zhao et al., 2023b)). These structural features strengthen interactions with enzyme active sites via hydrogen bonding and electrostatic effects. Ultrasonic-assisted TDES extraction further promotes matrix disruption, selective solubilisation, partial depolymerisation, and improved dispersion (IR, DLS, and zeta potential data) (Fan et al., 2026; Hong et al., 2021; Jia et al., 2021; Wang et al., 2022a; Xuejing et al., 2017; Zhan et al., 2022; Zhang et al., 2024; Zhang et al., 2023). Although these samples are crude polysaccharide fractions and minor contributions from co-extracted proteins or phenolics cannot be excluded, their effects appear limited. UD fractions, particularly SSPP-UD, had the highest polysaccharide content, the lowest impurity levels, and the strongest *α*-amylase and *α*-glucosidase inhibition (Table 3; Fig. 4E–F), whereas CE fractions contained more impurities and showed weaker activity, indicating that bioactivity is primarily attributable to polysaccharides.

Beyond structural attributes, the intrinsic antioxidant activity of crude polysaccharides can modulate *α*-amylase and *α*-glucosidase inhibition via two complementary mechanisms (Tan & Gan, 2016; Wang et al., 2024c). First, hydroxyl, carboxyl, and other polar groups can form hydrogen bonds and electrostatic or hydrophobic interactions with enzymes, thereby altering enzyme conformation, stabilising specific states, or hindering substrate binding (Li et al., 2024a; Wang et al., 2022b). Second, antioxidant moieties scavenge radicals while engaging enzyme active or peripheral sites, stabilising favourable conformations and local redox microenvironments (Qian et al., 2015; Wang et al., 2014a). Accordingly, UD-extracted polysaccharides leverage both structural and antioxidant features to strengthen enzyme inhibition and hypoglycemic potential.

In summary, the higher *α*-amylase and *α*-glucosidase inhibitory activities of SSPP-UD and BBP-UD arise from the combined effects of ultrasonic-assisted TDES extraction and fermentation. Key structural characteristics—abundant hydroxyl groups, lower molecular weight, smaller hydrodynamic size, modified zeta potential, and favourable monosaccharide profiles—together with enhanced antioxidant capacity, promote polysaccharide–enzyme interactions and support hypoglycemic activity. These results highlight extraction strategy as a determinant of polysaccharide bioactivity and provide a basis for further purification and mechanistic studies targeting postprandial glycemic control.

## 4 Conclusion

This study demonstrates that ultrasonic-assisted TDES extraction (UD) is an effective and sustainable approach for extracting bioactive crude polysaccharides from SSP. Under optimised conditions, UD increased yield and selectively enriched low-molecular-weight fractions, reflecting the synergistic effects of solvent design, ultrasonic cavitation, and enhanced matrix accessibility. Comparison with unfermented black beans (BB) revealed that fermentation alters monosaccharide composition and molecular weight distribution, thereby increasing polysaccharide solubility and bioactivity. Structural analyses further showed that UD-extracted polysaccharides (SSPP-UD) form small, well-dispersed aggregates enriched in functional components, including uronic acids and arabinose/xylose residues, which likely contribute to antioxidant and hypoglycemic activities. The reduced co-extraction of proteins and phenolic compounds likely reflects preferential interactions between TDES and polysaccharide hydroxyl/carboxyl groups, whereas cavitation enhances mass transfer and favours solubilisation of carbohydrate-rich domains. Functionally, SSPP-UD exhibited stronger radical-scavenging activity and potent dual inhibition of *α*-amylase and *α*-glucosidase, highlighting its potential as a functional food ingredient. Collectively, these findings clarify how extraction methodology and fermentation synergistically influence the structural and functional properties of legume-derived polysaccharides.

## 5 Future perspectives

Despite the demonstrated efficacy of ultrasonic-assisted TDES extraction, several limitations remain. The current TDES system contains ethylene glycol, which is not fully food compatible, and the molecular basis of fermentation-driven structural remodeling of SSP polysaccharides is unresolved. In addition, structure–activity relationships were inferred from crude fractions, and in vivo validation remains limited. These limitations highlight the need for further solvent optimisation and mechanistic studies.

Future research should address these gaps by:

(1) Developing food-grade solvents: replace ethylene glycol-based TDESs with fully food-compatible, non-toxic NADES while ensuring solvent recovery, stability, and regulatory compliance.
(2) Elucidate structure–function relationships: use purified polysaccharides with methylation analysis, 1D/2D NMR, and mass spectrometry to define glycosidic linkages, branching patterns, and conformational features.
(3) Expand functional validation: integrate cell-based assays, immunomodulatory profiling, *in vivo* studies, and omics approaches to identify molecular targets and validate bioactivities.
(4) Translate findings into functional foods: evaluate product stability, sensory attributes, digestibility, and regulatory requirements to support practical applications.
(5) Systematically evaluate fermentation effects: perform time-resolved monitoring of polysaccharide structure (molecular weight, glycosidic linkages, monosaccharide composition, and conformation) together with microbial and metabolomic profiling to elucidate the mechanisms of fermentation-driven modification.

Collectively, these strategies will enable sustainable production of high-performance polysaccharides, deepen mechanistic understanding, and facilitate their development as nutraceuticals and functional food ingredients.

## CRediT authorship contribution statement

**Mengjie Xu:** Writing – original draft, Investigation, Funding acquisition, Data curation. **Haobo Ma:** Methodology, Formal analysis, Conceptualization. **Yuan Yuan:** Software, Resources, Investigation. **Jing Guo:** Supervision, Software, Resources. **Jiao Kang:** Visualization, Supervision, Data curation. **Weirong Jie:** Validation, Supervision, Software, Resources. **Yunxi Yang:** Writing – review & editing, Writing – original draft, Project administration, Investigation.

## Declaration of competing interest

The authors declare that they have no known competing financial interests or personal relationships that could have appeared to influence the work reported in this paper.

## Data Availability

The data that has been used is confidential.

## Appendix A Supplementary data

Supplementary material Supplementary Information: Additional Figures, Tables, and Experimental Details.
