# Supplementary material for: Ultrasonic-assisted ternary deep eutectic solvent extraction of polysaccharides from fermented black beans (Dan-Dou-Chi): structural features, antioxidant, and hypoglycemic activities
Source: Food Chem X. 2026 Jun 15;37:104103. doi: 10.1016/j.fochx.2026.104103 (PMC13315902; doi:10.1016/j.fochx.2026.104103)
Supplement: Supplementary material — Supplementary Information: Additional Figures, Tables, and Experimental Details. [file mmc1.docx]

**Ultrasonic-Assisted Ternary Deep Eutectic Solvent Extraction of Polysaccharides from Fermented Black Beans (Dan-Dou-Chi): Structural Features, Antioxidant, and Hypoglycemic Activities**

Mengjie Xu ^a, c^, Haobo Ma ^d^, Yuan Yuan ^a^, Jing Guo ^a^, Jiao Kang ^a^, Weirong Jie ^a^, Yunxi Yang ^b*^

^a^ Department of Biological Sciences, XinZhou Normal University, Xinzhou, Shanxi 034000, China

^b^ Guangdong Provincial Engineering Technology Research Center for Innovative Drugs and Bioproducts, School of Pharmacy, Guangdong Medical University, Dongguan 523808, China

^c^ Shanxi Zhendong Pharmaceutical Co., Ltd. Changzhi, Shanxi 047100, China

^d^ Guangzhou University of Chinese Medicine faculty of Chinese medicine

*Corresponding author

*Address*: Guangdong Provincial Engineering Technology Research Center for Innovative Drugs and Bioproducts, School of Pharmacy, Guangdong Medical University, Dongguan 523808, China.

E-mail address: Xumj1019@126.com (Mengjie Xu), [2022064123@stu.gzucm.edu.cn,](mailto:2022064123@stu.gzucm.edu.cn,) (Haobo Ma), [Yy3439603585@qq.com](mailto:Yy3439603585@qq.com) (Yuan Yuan), 2366951655@qq.com (Jing Guo), [18335459508@163.com](mailto:18335459508@163.com) (Jiao Kang), jy2873124129@163.com (Weirong Jie), yunxiyang2009@gdmu.edu.cn (Yunxi Yang).

**List for** **supporting information**

**S1 Materials and Reagents**

**S2 Computational procedure for DFT calculations**

**S3 Hydrodynamic size and zeta potential analysis**

**S4 Monosaccharide Composition**

**S5 Molecular Weight (Mw)**

**S6 Fourier transform-infrared (FT-IR) spectroscopy and ultraviolet-visible (UV-Vis) absorption spectroscopy analysis**

**S7 *In vitro* antioxidant activity analysis**

**S8 Hypoglycemic activity assays**

**Fig. S1.** Single-factor variables on polysaccharide yield from SSP.................................8

**Fig. S2.** Diagnostic plots for the quadratic regression model of SSP polysaccharide yield............................................................................................................................................9

**Fig. S3**. Contour plots for the effects of extraction duration, the water content in DES, solvent-to-solid ratio, and ultrasonic temperature on SSPP yields..................................10

**Fig. S4.** SEM images showing the morphology of SSPPs and BBPs obtained using different extraction methods at magnifications of 2000×.................................................11

**Table S1.** Experimental matrix for CE, UW, BDES-UAE, and TDES-UAE extraction conditions................................................................................................................................12

**Table S2.** Variables studied in the experimental design with their respective coded and real levels.................................................................................................................................12

**Table S3.** Response surface experimental design and results....................................12-14

**Table S4.** Physicochemical properties (viscosity, density, and pH) of different DES samples at various temperatures with statistical grouping.............................................................................................................................14-17

**Table S5.** Concentration-dependent antioxidant activities of different samples evaluated by DPPH radical scavenging, ABTS•⁺ radical cation scavenging, hydroxyl radical scavenging, and ferric reducing antioxidant power (FRAP) assays with statistical comparisons......................................................................................................17-26

**Table S6.** Concentration-dependent inhibitory activities of different samples against *α*-amylase and *α*-glucosidase with statistical comparisons.........................................26-30

**Table S7.** IC_50_ values of SSPP and BBP fractions for *α*‑amylase and *α*‑glucosidase inhibition, with 95% confidence intervals and R^2^ of the dose-response fits..................31

**S1 Materials and Reagents**

*Semen Sojae Praeparatum* (SSP) and its unfermented precursor, black beans (BB), were procured from Beijing Tongrentang Co., Ltd. (Beijing, China), with the botanical identity authenticated by Professor Jun Yin, Shenyang Pharmaceutical University. Voucher specimens were deposited in the Guangdong Provincial Key Laboratory of Natural Drugs Research and Development, Guangdong Medical University (Dongguan, China) under the numbers 20250111 Tibet-01 (SSP) and 20250111 Tibet-03 (BB). All reagents were analytical grade unless specified, sourced as follows: choline chloride (ChCl, ≥98%), 1,3-butanediol (1,3-Buta, ≥99%), 1,4-butanediol (1,4-Buta, ≥98%), 1,2-propanediol (1,2-PG, ≥99%), 1,3-propanediol (1,3-PG, ≥98%), ethylene glycol (EG, ≥98%), glycerol (Gly, ≥99%), D,L-malic acid (MA, ≥99.5%), oxalic acid (OA, ≥99.5%), D,L-tartaric acid (TA, ≥99%), citric acid (CA, ≥99.5%), D,L-lactic acid (LA, ≥99.5%), along with anhydrous ethanol, trichloroacetic acid, sodium chloride, ferric chloride hexahydrate, ferrous sulfate heptahydrate, and salicylic acid, were obtained from Macklin Biochemical Co., Ltd. (Shanghai, China). D-glucose and monosaccharide standards (fucose, rhamnose, arabinose, galactose, mannose, xylose, fructose, and ribose) were obtained from Borui Sugar Biotechnology Co., Ltd.. Ltd. Trifluoroacetic acid (Acros Organics, Fair Lawn, NJ, USA), sodium hydroxide (Alfa Aesar, Ward Hill, MA, USA), and sodium acetate (Thermo Fisher Scientific, Waltham, MA, USA) were used for the chromatography. Antioxidant and enzyme inhibition assay reagents—1,1-diphenyl-2-picrylhydrazyl (DPPH), 2,2′-azinobis-(3-ethylbenzthiazoline-6-sulphonate) (ABTS), 2,4,6-tripyridyl-s-triazine (TPTZ), acarbose, *α*-glucosidase, *α*-amylase, 3,5-dinitrosalicylic acid, and *p*-nitrophenyl-D-glucoside (*p*NPG)—were purchased from Sigma-Aldrich Co. (St. Louis, MO, USA). Deionized water was purified using a Milli-Q water purification system (Millipore, Bedford, MA, USA).

**S2 Computational procedure for DFT calculations**

All quantum chemical calculations were performed using the Gaussian 16 software package (Revision C.01) (Frisch et al., 2010). The molecular geometries were fully optimized at the B3LYP level of theory (Becke & Axel, 1993; Lee et al., 1988; Miehlich et al., 1989) in conjunction with the def2-DZVP basis set (Schafer et al., 1992; Weigend & Ahlrichs, 2005). Frequency calculations were subsequently carried out at the same level to confirm that all optimized structures correspond to true minima on the potential energy surface, as indicated by the absence of imaginary frequencies.

To evaluate intermolecular interactions within the DES system, the interaction energy was calculated using the supermolecular approach according to:

$$\text{E}_{\text{int}}\text{=}\text{E}_{\text{complex}}\text{-}\sum\text{E}_{\text{monomers}}$$

Where $\text{E}_{\text{complex}}$and $\text{E}_{\text{monomers}}$represent the total energies of the optimized complex and the isolated components, respectively. This approach is widely used to estimate the strength of noncovalent interactions such as hydrogen bonding. Basis set superposition error (BSSE) correction was not considered, as the present calculations were intended for qualitative comparison of hydrogen-bonding interactions rather than high-precision energy evaluation.

Key hydrogen-bond distances were extracted from the optimized geometries to further characterize the hydrogen-bonding network within the DES cluster. The electrostatic potential (ESP) surfaces were calculated to analyze the charge distribution and potential reactive sites of the molecular systems. ESP analyses were performed using the Multiwfn program (version 3.8) (Lu & Chen, 2012). The resulting ESP-mapped molecular surfaces were visualized using Visual Molecular Dynamics (VMD, version 1.9.4) (Humphrey et al., 1996).

**S3 Hydrodynamic size and zeta potential analysis**

The hydrodynamic diameter (Dh) and zeta potential of polysaccharide aggregates in aqueous suspension were characterized using a dynamic light scattering (DLS) instrument (Malvern Nano-S90, Malvern Instruments, Worcestershire, UK) (Geng et al., 2024). For sample pretreatment, polysaccharide solutions were prepared at an initial concentration of 10 mg/mL, vortexed thoroughly to ensure homogeneous dispersion, and centrifuged at 6500 rpm for 5 min to remove large aggregates. For hydrodynamic size measurement, 200 μL of the supernatant was diluted 10-fold with deionized water to optimize the concentration for DLS analysis and minimize multiple scattering effects. Zeta potential measurements were performed using the same pretreated solution under ambient temperature conditions. The zeta potential detection mode of the DLS instrument was used to quantify the electrophoretic mobility of the aggregates, which was subsequently converted to zeta potential values using Smoluchowski’s equation. All analyses were performed in triplicate to ensure reproducibility. Hydrodynamic size results are reported as intensity-weighted Dh in micrometers (μm), and zeta potential values are presented as absolute values in millivolts (mV). All data are expressed as mean ± standard deviation.

**S4 Monosaccharide Composition**

Monosaccharide profiling of the polysaccharides was conducted via ion chromatography using an ICS5000+ system (Thermo Fisher Scientific, Waltham, MA, USA) equipped with a Dionex Carbopac™ PA10 column (4 × 250 mm), following a modified protocol (Geng et al., 2024). Briefly, 5 mg of each polysaccharide sample was placed in a sealed reaction tube and combined with 2 mL of 3 M trifluoroacetic acid (TFA) for hydrolysis at 120 °C for 3 h. After hydrolysis, residual TFA was removed by repeated evaporation under a stream of nitrogen gas to avoid interference with subsequent analysis. The hydrolyzed residues were redissolved in distilled water, and the resulting solution was filtered through a 0.45-μm membrane to remove particulate matter. For chromatographic analysis, 25 μL of the filtered solution was injected into the system, and the column was maintained at 30 °C. Elution was performed using three mobile phases: (A) ultrapure water, (B) 500 mM NaOH containing 50 mM NaOAc, and (C) 20 mM NaOH solution. Gradient conditions were optimized to achieve baseline separation of standard monosaccharides, including fucose, rhamnose, arabinose, galactose, glucose, mannitose, xylose, fructose, and ribose, to ensure accurate quantification of each component in the samples.

**S5 Molecular Weight (Mw)**

The molecular weight (Mw) of the polysaccharides was determined by high-performance gel permeation chromatography (HPGPC) using a Thermo U3000 chromatograph coupled with a Shimadzu RI-20A refractive index detector and BRT 105-103-101 tandem gel columns (8 × 300 mm). Dextran standards (P5, P10, P20, P50, P100, P200, P400, P800; SHOWA DENKO, ≥97% purity) were used for calibration.

The mobile phase was a 0.5 M NaCl solution, prepared by dissolving 58.44 g of NaCl in ultrapure water, adjusting the final volume to 2 L, sonicating for 10 min, and filtering it through a 0.22 μm membrane. The solution was stored at room temperature and used within three days. Standard solutions were prepared by dissolving 5 mg of each dextran standard in 1 mL of the mobile phase. Sample solutions were prepared by dissolving 5 mg of polysaccharide in 1 mL of the mobile phase, sonicating for 10 min, centrifuging at 12000 rpm for 10 min, and filtering the supernatant through a 0.22 μm aqueous membrane. The chromatographic conditions were as follows: mobile phase, 0.5 M NaCl; flow rate, 0.7 mL/min; column temperature, 40 °C; injection volume, 50 μL; detection via refractive index detector. Calibration curves relating the logarithm of molecular weight (LgMp, LgMw, and LgMn) to retention time were established using dextran standards. The peak molecular weight (Mp), weight-average molecular weight (Mw), and number-average molecular weight (Mn) of each elution fraction were calculated based on the corresponding calibration equations. The number of chromatographic peaks for each sample was determined from the HPGPC chromatograms, and the relative percentage of each peak was calculated by peak area integration followed by normalization. In addition, the overall molecular weight parameters (Mw and Mn) of each polysaccharide sample were obtained by integrating the chromatographic peaks and calculating area-weighted averages according to peak area distribution. The polydispersity index (Mw/Mn) was used to evaluate the molecular weight distribution. It should be noted that, due to the characteristics of the refractive index (RI) detector, the signal intensity is dependent on the solute concentration and its distribution along the elution profile, which may result in differences in peak intensity among samples with different molecular weight distributions. All measurements were performed in triplicate, and the results are expressed as mean ± standard deviation.

**S6 Fourier transform-infrared (FT-IR) spectroscopy and ultraviolet-visible (UV-Vis) absorption spectroscopy analysis**

Fourier transform-infrared spectroscopy (FT-IR) was used to identify the functional groups of the polysaccharides using an FT-IR spectrophotometer (Thermo Fisher, USA) (Wang et al., 2014). A comprehensive mixture of 1 mg freeze-dried polysaccharide and 150 mg KBr was prepared, and the mixture was pressed into pellets. Spectra were recorded over the wavenumber range of 400–4000 cm⁻¹.

Ultraviolet-visible (UV–Vis) absorption spectroscopy was conducted using a Cary 60 UV-Vis spectrophotometer (Agilent, USA) (Geng et al., 2024) to detect potential residual impurities. Polysaccharide samples were dissolved in deionized water to a final concentration of 1 mg/mL, and the solutions were filtered through a 0.22 μm membrane before analysis. Absorption spectra were collected over a wavelength range of 200–800 nm, with deionized water serving as a blank control to correct for the background absorption. This method allows the identification of characteristic absorption peaks, with proteins showing a peak at approximately 280 nm (Mohammed et al., 2020).

**S7 *In vitro* antioxidant activity analysis**

**S7-1 DPPH radical scavenging activity**

The DPPH radical-scavenging potential of the polysaccharide samples was determined using a modified protocol based on Zhang et al. (2022). Samples were dissolved in distilled water to prepare a series of concentrations (0.1, 0.2, 0.3, 0.4, 0.5, 1.0, 1.5, 2.0, 2.5, and 3.0 mg/mL). Each sample solution (1.5 mL) was mixed with 1.5 mL of a 0.2 mM DPPH solution in 95% ethanol. Ascorbic acid (Vc) at the corresponding concentrations was used as the positive control. The reaction mixtures were incubated in the dark at room temperature for 30 min to allow the reaction to reach equilibrium. The absorbance was measured at 517 nm using a UV-Vis spectrophotometer (UV-2600; Shimadzu, Japan). The scavenging rate was calculated using the following equation:

$\text{DPPH radical scavenging activity }\text{(}\text{\%}\text{) = }\left[ \text{1-}\frac{\left( \text{As}\text{-}\text{Aos} \right)}{\text{Ao}} \right]\text{×100\%}$

Where $\text{Ao}$ is the absorbance of the blank control (DPPH solution without sample), $\text{As}$ is the absorbance of the sample mixed with DPPH solution, and $\text{Aos}$ is the absorbance of the sample mixed with 95% ethanol (without DPPH). All measurements were performed in triplicate, and the results are expressed as mean ± standard deviation.

**S7-2 ABTS radical scavenging activity**

The ability of the samples to scavenge 2,2′-azinobis-(3-ethylbenzothiazoline-6-sulphonate) (ABTS^•^⁺) radicals was determined using a modified method described by Saravanakumar et al. (2021). The ABTS^•^⁺ stock solution was prepared by reacting 7 mM ABTS with 2.45 mM potassium persulfate and stored in the dark at room temperature for 16 h. The solution was then diluted with 60% ethanol to achieve an absorbance of 1.00 ± 0.02 at 734 nm wavelength. Sample solutions (0.5 mL) at varying concentrations (0.1, 0.2, 0.3, 0.4, 0.5, 1.0, 1.5, 2.0, 2.5, and 3.0 mg/mL) were mixed with 3.0 mL of the diluted ABTS^•^⁺ solution and incubated in the dark for 20 min at room temperature. Ascorbic acid (Vc) was used as the positive control. The absorbance was measured at 734 nm using UV-Vis spectrophotometry. The scavenging activity was calculated using the following equation:

$\text{ABTS }\text{redical scavenging}\text{ }\text{activity}\text{ }\text{(}\text{\%}\text{) = }\left[ \text{1-}\frac{\left( \text{As}\text{-}\text{Aos} \right)}{\text{Ao}} \right]\text{×100\%}$

where $\text{Ao}$ is the absorbance of the blank control (ABTS^•^⁺ solution without sample), $\text{As}$ is the absorbance of the sample mixed with ABTS^•^⁺ solution, and $\text{Aos}$ is the absorbance of the sample mixed with 60% ethanol (without ABTS•⁺). All assays were performed in triplicate, and the results are expressed as mean ± standard deviation.

**S7-3 Hydroxyl radical scavenging capacity**

The hydroxyl radical scavenging capacities of the samples were assessed using the modified approach described by Chen and Huang (2019). Briefly, 1 mL of the sample solution at various concentrations (0.5, 1.0, 1.5, 2.0, 2.5, and 3.0 mg/mL) was added to a stoppered test tube, followed by the sequential addition of 1 mL of 9×10^-3^ mol/L FeSO_4_ solution, 1 mL of 9×10^-3^ mol/L salicylic acid-ethanol solution, and 1 mL of 9×10^-3^ mol/L H_2_O_2_ solution. The mixture was vortexed to ensure homogeneity and incubated at 37 °C for 30 min. The absorbance was measured at 510 nm using a UV-Vis spectrophotometer (UV-2600, Shimadzu, Japan). The scavenging activity was calculated using the following equation:

$\text{Hydroxyl }\text{redical scavenging}\text{ }\text{activity}\text{ }\text{(}\text{\%}\text{) = }\left[ \text{1-}\frac{\left( \text{As}\text{-}\text{Aos} \right)}{\text{Ao}} \right]\text{×100\%}$

where $\text{Ao}$ denotes the absorbance of the blank control (reaction mixture without sample), $\text{As}$ is the absorbance of the sample-containing reaction mixture, and $\text{Aos}$ is the absorbance of the sample mixture without H_2_O_2_ (to account for background absorbance). All measurements were performed in triplicate, and the results are presented as mean ± standard deviation.

**S7-4 Ferric reducing antioxidant power (FRAP) assay**

The FRAP assay was performed with minor modifications, as described by González-Centeno et al. (2012). The working FRAP reagent was prepared by mixing 20 mM FeCl_3_·6H_2_O aqueous solution, 10 mM 2,4,6-tripyridyl-s-triazine (TPTZ) in 0.04 M HCl, and 300 mM acetate buffer (pH 3.6) at a ratio of 1:1:10 (v/v/v). In a 96-well microplate, 190 μL of the FRAP reagent was mixed with 10 μL of the sample solution at varying concentrations (0.5, 1.0, 1.5, 2.0, 2.5, and 3.0 mg/mL) and incubated at room temperature for 20 min. Deionized water was used as the blank control, and ascorbic acid (Vc) was used as the positive control. Absorbance was measured at 593 nm using a microplate reader (model 680; Bio-Rad, USA). A standard curve was generated using FeSO_4_·7H_2_O solutions (0.003125-0.15 μmol/mL), and the amount of Fe^2+^ equivalents in the sample was calculated based on the absorbance of the sample. The final FRAP values were expressed as micromoles of Fe^2+^ equivalents per milliliter of the sample (μmol/mL). All measurements were performed in triplicate, and the results are reported as mean ± standard deviation.

**S8 Hypoglycemic activity assays**

**S8-1 *α*-Amylase inhibitory assay**

The *α*-amylase inhibitory potential of the test samples was evaluated using a refined protocol adapted from Wang et al. (2018a). Briefly, 0.5 mL aliquots of polysaccharide solutions at escalating concentrations (0.5-3.0 mg/mL) were pre-incubated with 1 mL of porcine pancreatic *α*-amylase solution (2.5 U/mL) at 40°C for 10 min to facilitate enzyme-substrate complexation. Subsequently, 1 mL of soluble starch solution (1.0% w/v, prepared in distilled water) was added to initiate the enzymatic reaction, which was performed at 40 °C for 15 min. The reaction was quenched by adding 3 mL of 3,5-dinitrosalicylic acid (DNS) reagent, followed by boiling for 5 min to induce chromogenic development. After cooling to ambient temperature, the reaction mixture was diluted to 20 mL with deionized water, and the absorbance was measured at 540 nm using UV-Vis spectrophotometry. The *α*-amylase inhibitory efficacy was calculated using the following equation:

$\text{α}\text{-}\text{amylase inhibitory rate}\text{(}\text{\%}\text{) = }\left[ \text{1-}\frac{\left( \text{ }\text{A}_{\text{sample}}\text{-}\text{A}_{\text{sample}\text{ }\text{blank}} \right)}{\left( \text{A}_{\text{control}}\text{-}\text{A}_{\text{reagent}\text{ }\text{blank}} \right)} \right]\text{×100\%}$

Where $\text{A}_{\text{sample}}$ denotes the absorbance of the test sample with enzyme, $\text{A}_{\text{sample}\text{ }\text{blank}}$ denotes the absorbance of the sample without enzyme, $\text{A}_{\text{control}}$ denotesthe absorbance of the control group without samples, and $\text{A}_{\text{reagent}\text{ }\text{blank}}$ denotes the absorbance of the blank control group without samples and enzyme.

**S8-2 *α-*Glucosidase inhibitory assay**

*α*-Glucosidase inhibitory activity was assessed using a modified protocol based on previously established methodologies (Wang et al., 2018b). Briefly, 50 μL of polysaccharide solutions at varying concentrations (0.5-3 mg/mL) were preincubated with 100 μL of *α*-glucosidase (0.5 U/mL, prepared in 200 mM phosphate buffer, pH 7.0) at 37 °C for 10 min. The enzymatic reaction was initiated by adding 100 μL of *p*-nitrophenyl-*α*-D-glucopyranoside (*p*NPG, 5 mM in phosphate buffer) and incubated at 37 °C for 20 min. The reaction was terminated by the addition of 0.2 M sodium carbonate solution, and the absorbance was measured at 405 nm using a microplate reader (Multiskan GO; Thermo Fisher Scientific, USA). The inhibitory potency was calculated using the following equation:

$\text{α-}\text{glucosidase}\text{ inhibitory rate}\text{(}\text{\%}\text{) = }\left[ \text{1-}\frac{\left( \text{ }\text{A}_{\text{sample}}\text{-}\text{A}_{\text{sample}\text{ }\text{blank}} \right)}{\left( \text{A}_{\text{control}}\text{-}\text{A}_{\text{reagent}\text{ }\text{blank}} \right)} \right]\text{×100\%}$

Where $\text{A}_{\text{sample}}$ represents the absorbance of the test sample with enzyme, $\text{A}_{\text{sample}\text{ }\text{blank}}$ represents the absorbance of the sample without enzyme, and $\text{A}_{\text{control}}$ represents the absorbance of the control group without samples, and $\text{A}_{\text{reagent}\text{ }\text{blank}}$ denotes the absorbance of the blank control group without samples and enzyme.

For all hypoglycemic activity assays, acarbose was used as a positive control, and all experiments were conducted in triplicate.

**Fig. S1.** Single-factor variables on polysaccharide yield from SSP: (A) the water content in DES, (B) extraction duration, (C) solvent-to-solid ratio, and (D) ultrasonic temperature. Error Bars represent the standard deviation of three individual samples.

**Fig. S2.** Diagnostic plots for the quadratic regression model of SSP polysaccharide yield: (A) normal probability plot of residuals, (B) predicted versus actual yields, (C) residuals versus predicted values, and (D) residuals versus experimental run order.

**Fig. S3**. Contour plots for the effects of extraction duration, the water content in DES, solvent-to-solid ratio, and ultrasonic temperature on SSPP yields.

**Fig. S4.** SEM images showing the morphology of SSPPs and BBPs obtained using different extraction methods at magnifications of 2000×.

**Table S1.** Experimental matrix for CE, UW, BDES-UAE, and TDES-UAE extraction conditions.

| **Method** | **Solvent system** | **Water content** | **Liquid-to-solid ratio** | **Temperature** | **Time** | **Ultrasound** |
| --- | --- | --- | --- | --- | --- | --- |
| CE | Deionized water | 100% | 30 mL/g | Boiling water bath | 6 h | No |
| UW | Deionized water | 100% | 30 mL/g | 80 °C | 60 min | Yes |
| BDES-UAE (screening) | DES-1~DES-6 | 30% | 30 mL/g | 80 °C | 60 min | Yes |
| TDES-UAE (screening) | DES-7~DES-21 | 30% | 30 mL/g | 80 °C | 60 min | Yes |
| UD-9 (non-optimized) | DES-9 | 30% | 30 mL/g | 80 °C | 60 min | Yes |
| UD-9 (optimized) | DES-9 | 35% | 30 mL/g | 65 °C | 79 min | Yes |

**Table S2.** Variables studied in the experimental design with their respective coded and real levels.

| Variable | BBD | | | |
| --- | --- | --- | --- | --- |
|  | Symbol | Coded levels | | |
|  |  | -1 | 0 | 1 |
| Extraction duration | A | 60 | 75 | 90 |
| The water content in DES | B | 30 | 40 | 50 |
| Solvent-to-solid ratio | C | 25 | 30 | 35 |
| Ultrasonic temperature | D | 60 | 70 | 80 |

**Table S3.** Response surface experimental design and results.

| Run | Factor A | Factor B | Factor C | Factor D | Polysaccharide yields  (mg/g) |
| --- | --- | --- | --- | --- | --- |
|  | Extraction duration (min) | The water content in DES (%) | Solvent-to-solid ratio (mL/g) | Ultrasonic temperature (°C) |  |
| 1 | 60 | 40 | 30.00 | 60 | 13.93 ± 1.20 |
| 2 | 75 | 30 | 30.00 | 80 | 75.83 ± 1.72 |
| 3 | 75 | 30 | 35.00 | 70 | 81.86 ± 1.48 |
| 4 | 75 | 40 | 30.00 | 70 | 108.54 ± 0.96 |
| 5 | 75 | 40 | 25.00 | 60 | 79.88 ± 1.57 |
| 6 | 75 | 50 | 30.00 | 80 | 45.56 ± 1.38 |
| 7 | 60 | 30 | 30.00 | 70 | 61.94 ± 1.46 |
| 8 | 60 | 40 | 30.00 | 80 | 79.57 ± 2.36 |
| 9 | 75 | 40 | 25.00 | 80 | 41.27 ± 2.41 |
| 10 | 60 | 40 | 35.00 | 70 | 7.16 ± 0.82 |
| 11 | 75 | 40 | 35.00 | 80 | 75.65 ± 0.83 |
| 12 | 75 | 50 | 30.00 | 60 | 42.77 ± 1.79 |
| 13 | 90 | 40 | 30.00 | 60 | 92.61 ± 1.62 |
| 14 | 75 | 40 | 30.00 | 70 | 107.25 ± 1.17 |
| 15 | 90 | 30 | 30.00 | 70 | 65.02 ± 0.25 |
| 16 | 75 | 50 | 25.00 | 70 | 34.21 ± 2.03 |
| 17 | 90 | 40 | 35.00 | 70 | 68.90 ± 2.05 |
| 18 | 75 | 40 | 30.00 | 70 | 108.27 ± 2.25 |
| 19 | 60 | 40 | 25.00 | 70 | 63.37 ± 2.22 |
| 20 | 90 | 40 | 25.00 | 70 | 10.34 ± 0.57 |
| 21 | 90 | 40 | 30.00 | 80 | 9.29 ± 0.76 |
| 22 | 75 | 30 | 30.00 | 60 | 99.87 ± 1.73 |
| 23 | 75 | 40 | 35.00 | 60 | 55.49 ± 1.90 |
| 24 | 90 | 50 | 30.00 | 70 | 19.98 ± 2.36 |
| 25 | 60 | 50 | 30.00 | 70 | 16.50 ± 1.45 |
| 26 | 75 | 30 | 25.00 | 70 | 72.07 ± 2.40 |
| 27 | 75 | 50 | 35.00 | 70 | 33.10 ± 2.16 |
| 28 | 75 | 40 | 30.00 | 70 | 107.97 ± 1.46 |
| 29 | 75 | 40 | 30.00 | 70 | 108.33 ± 2.22 |

**Table S4.** Physicochemical properties (viscosity, density, and pH) of different DES samples at various temperatures with statistical grouping*.

| Temperature (K) | Sample | Viscosity (mean ± SD, n = 3) | Viscosity group | Density (mean ± SD, n = 3) | Density group | pH  (mean ± SD, n = 3) | pH group |
| --- | --- | --- | --- | --- | --- | --- | --- |
| 303.15 | DES-7 | 25.7±1.73 | c | 1.17±0.18 | a | 2.39±0.06 | b |
|  | DES-8 | 24.35±1.45 | cd | 1.15±0.04 | a | 1.11±0.14 | c |
|  | DES-9 | 30.66±0.71 | bc | 1.19±0.02 | a | 2.11±0.01 | b |
|  | DES-10 | 39.42±2.26 | a | 1.23±0.11 | a | 2.20±0.17 | b |
|  | DES-11 | 26.27±1.22 | c | 1.15±0.03 | a | 2.90±0.22 | a |
|  | DES-12 | 23.68±0.85 | cd | 1.14±0.08 | a | 2.44±0.14 | b |
|  | DES-13 | 20.03±0.19 | de | 1.13±0.13 | a | 1.19±0.09 | c |
|  | DES-14 | 27.32±0.95 | c | 1.17±0.13 | a | 2.24±0.19 | b |
|  | DES-15 | 27.84±1.15 | bc | 1.18±0.03 | a | 2.24±0.22 | b |
|  | DES-16 | 14.58±1.19 | e | 1.11±0.05 | a | 2.96±0.09 | a |
|  | DES-17 | 24.72±1.91 | cd | 1.14±0.01 | a | 2.45±0.03 | b |
|  | DES-18 | 21.58±0.82 | d | 1.1±0.08 | a | 1.40±0.01 | c |
|  | DES-19 | 29.68±0.74 | bc | 1.15±0.07 | a | 2.26±0.13 | b |
|  | DES-20 | 31.38±1.01 | b | 1.17±0.06 | a | 2.35±0.12 | b |
|  | DES-21 | 16.56±1.35 | e | 1.1±0.11 | a | 3.13±0.25 | a |
| 313.15 | DES-7 | 17.63±1.48 | d | 1.16±0.06 | a | 2.29±0.23 | b |
|  | DES-8 | 15.77±1.06 | de | 1.15±0.08 | a | 1.03±0.18 | c |
|  | DES-9 | 25.36±0.75 | b | 1.19±0.09 | a | 2.02±0.20 | b |
|  | DES-10 | 29.52±0.65 | a | 1.21±0.1 | a | 2.13±0.11 | b |
|  | DES-11 | 21.44±1.16 | c | 1.14±0.02 | a | 2.53±0.20 | ab |
|  | DES-12 | 15.74±1.14 | de | 1.14±0.01 | a | 2.35±0.07 | b |
|  | DES-13 | 12.57±1.4 | e | 1.12±0.01 | a | 1.13±0.02 | c |
|  | DES-14 | 21.44±1.33 | c | 1.16±0.06 | a | 2.16±0.18 | b |
|  | DES-15 | 19.63±1.5 | cd | 1.17±0.02 | a | 2.20±0.20 | b |
|  | DES-16 | 12.65±0.83 | e | 1.11±0.07 | a | 2.72±0.09 | ab |
|  | DES-17 | 16.4±1.37 | de | 1.13±0.03 | a | 2.40±0.03 | b |
|  | DES-18 | 14.6±0.75 | de | 1.1±0.11 | a | 1.28±0.03 | c |
|  | DES-19 | 22.61±0.94 | bc | 1.14±0.07 | a | 2.20±0.20 | b |
|  | DES-20 | 24.69±1.15 | bc | 1.16±0.04 | a | 2.31±0.30 | b |
|  | DES-21 | 13.54±1.28 | e | 1.09±0.1 | a | 2.99±0.20 | a |
| 323.15 | DES-7 | 15.34±0.85 | c | 1.16±0.03 | a | 2.18±0.14 | bc |
|  | DES-8 | 12.48±1.22 | cd | 1.41±0.28 | a | 0.89±0.30 | d |
|  | DES-9 | 17.74±1.11 | bc | 1.19±0.19 | a | 1.93±0.05 | c |
|  | DES-10 | 26.62±1.68 | a | 1.21±0.1 | a | 2.07±0.14 | bc |
|  | DES-11 | 18.53±1.34 | bc | 1.12±0.11 | a | 2.47±0.10 | b |
|  | DES-12 | 13.55±0.94 | cd | 1.13±0.01 | a | 2.23±0.23 | bc |
|  | DES-13 | 10.48±1.33 | d | 1.12±0.11 | a | 1.02±0.06 | d |
|  | DES-14 | 16.58±1.01 | bc | 1.16±0.05 | a | 2.08±0.18 | bc |
|  | DES-15 | 15.56±1.17 | c | 1.16±0.12 | a | 2.11±0.22 | bc |
|  | DES-16 | 9.19±0.95 | d | 1.1±0.02 | a | 2.50±0.03 | ab |
|  | DES-17 | 14.77±0.94 | c | 1.12±0.02 | a | 2.29±0.02 | bc |
|  | DES-18 | 11.33±0.83 | d | 1.09±0.11 | a | 1.18±0.10 | d |
|  | DES-19 | 17.65±1.19 | bc | 1.13±0.01 | a | 2.18±0.19 | bc |
|  | DES-20 | 19.46±0.67 | b | 1.16±0.06 | a | 2.19±0.19 | bc |
|  | DES-21 | 10.45±1.28 | d | 1.08±0.1 | a | 2.96±0.04 | a |
| 333.15 | DES-7 | 12.06±0.98 | bc | 1.15±0.01 | a | 2.12±0.04 | bc |
|  | DES-8 | 10±1.08 | c | 1.13±0.1 | a | 0.77±0.06 | d |
|  | DES-9 | 13.33±1.07 | b | 1.18±0.11 | a | 1.89±0.10 | c |
|  | DES-10 | 18.74±0.95 | a | 1.21±0.1 | a | 1.92±0.17 | c |
|  | DES-11 | 14.37±1.35 | b | 1.09±0.08 | a | 2.40±0.15 | b |
|  | DES-12 | 11.36±1.18 | bc | 1.13±0.07 | a | 2.17±0.11 | bc |
|  | DES-13 | 8.89±1.02 | c | 1.11±0.11 | a | 0.94±0.04 | d |
|  | DES-14 | 12.71±0.73 | bc | 1.15±0.01 | a | 1.97±0.11 | c |
|  | DES-15 | 13.06±1.61 | bc | 1.16±0.06 | a | 1.96±0.17 | c |
|  | DES-16 | 7.67±0.41 | c | 1.09±0.1 | a | 2.46±0.03 | b |
|  | DES-17 | 12.82±0.87 | bc | 1.12±0.05 | a | 2.25±0.23 | bc |
|  | DES-18 | 9.73±0.85 | c | 1.06±0.07 | a | 1.09±0.05 | d |
|  | DES-19 | 13.86±0.87 | b | 1.13±0.05 | a | 2.14±0.12 | bc |
|  | DES-20 | 15.7±0.86 | ab | 1.15±0.07 | a | 2.08±0.26 | bc |
|  | DES-21 | 9.23±1.12 | c | 1.07±0.11 | a | 2.92±0.05 | a |
| 343.15 | DES-7 | 9.69±0.4 | b | 1.15±0.01 | a | 2.05±0.18 | bc |
|  | DES-8 | 8.69±1.38 | bc | 1.13±0.1 | a | 0.69±0.07 | d |
|  | DES-9 | 10.83±1.15 | ab | 1.17±0.08 | a | 1.81±0.09 | c |
|  | DES-10 | 12.84±1.08 | a | 1.19±0.07 | a | 1.84±0.10 | c |
|  | DES-11 | 11.63±1.24 | ab | 1.08±0.05 | a | 2.39±0.14 | b |
|  | DES-12 | 9.34±0.22 | bc | 1.12±0.09 | a | 2.13±0.10 | bc |
|  | DES-13 | 7.72±1.28 | bc | 1.11±0.1 | a | 0.83±0.16 | d |
|  | DES-14 | 9.66±1.29 | bc | 1.15±0.02 | a | 1.92±0.25 | c |
|  | DES-15 | 10.45±1.27 | ab | 1.15±0.06 | a | 1.88±0.00 | c |
|  | DES-16 | 6.54±0.5 | c | 1.08±0.09 | a | 2.44±0.02 | ab |
|  | DES-17 | 10.4±1.17 | ab | 1.11±0.08 | a | 2.20±0.22 | bc |
|  | DES-18 | 8.85±0.96 | bc | 1.04±0.14 | a | 0.90±0.18 | d |
|  | DES-19 | 11.64±0.78 | ab | 1.12±0 | a | 2.08±0.18 | bc |
|  | DES-20 | 12.57±0.65 | ab | 1.15±0.05 | a | 1.91±0.01 | c |
|  | DES-21 | 7.75±1.33 | bc | 1.07±0.11 | a | 2.85±0.01 | a |
| 353.15 | DES-7 | 8.58±1.29 | b | 1.14±0.01 | a | 1.96±0.19 | bc |
|  | DES-8 | 7.83±0.82 | b | 1.12±0.09 | a | 0.57±0.07 | d |
|  | DES-9 | 8.88±1.09 | ab | 1.16±0.07 | a | 1.69±0.10 | c |
|  | DES-10 | 11.86±1.07 | a | 1.19±0.17 | a | 1.78±0.24 | c |
|  | DES-11 | 9.67±0.63 | ab | 1.08±0.08 | a | 2.33±0.14 | b |
|  | DES-12 | 8.23±1.06 | b | 1.11±0.11 | a | 2.06±0.07 | bc |
|  | DES-13 | 6.71±0.73 | b | 1.09±0.1 | a | 0.72±0.17 | d |
|  | DES-14 | 8.33±0.37 | b | 1.14±0.03 | a | 1.87±0.12 | c |
|  | DES-15 | 8.7±0.63 | b | 1.14±0.04 | a | 1.86±0.12 | c |
|  | DES-16 | 5.77±0.97 | b | 1.08±0.06 | a | 2.41±0.02 | ab |
|  | DES-17 | 8.69±1.36 | b | 1.1±0.09 | a | 2.11±0.13 | bc |
|  | DES-18 | 7.38±1.44 | b | 1.02±0.08 | a | 0.79±0.24 | d |
|  | DES-19 | 9.02±0.99 | ab | 1.11±0.05 | a | 2.03±0.13 | bc |
|  | DES-20 | 10.43±1.15 | ab | 1.14±0.1 | a | 1.89±0.01 | c |
|  | DES-21 | 5.93±0.95 | b | 1.06±0.07 | a | 2.81±0.03 | a |

*Values are expressed as mean ± SD (n = 3). For each temperature, statistical comparisons among different samples were performed using one-way ANOVA followed by Tukey’s multiple comparison test. Different lowercase letters indicate significant differences among samples at the same temperature within each physicochemical property (*p* < 0.05). The same letters indicate no significant difference.

**Table S5.** Concentration-dependent antioxidant activities of different samples evaluated by DPPH radical scavenging, ABTS•⁺ radical cation scavenging, hydroxyl radical scavenging, and ferric reducing antioxidant power (FRAP) assays with statistical comparisons*.

| Assay | Concentration | Sample | Inhibition rate (%) | Group | Assay | Concentration | Sample | Inhibition rate (%) | Group |
| --- | --- | --- | --- | --- | --- | --- | --- | --- | --- |
| DPPH radical scavenging | 0.1 | Vc | 22.81 ± 0.19 | a | Hydroxyl radical scavenging | 0.5 | Vc | 58.14 ± 0.55 | a |
| DPPH radical scavenging | 0.1 | SSPP-CE | 2.03 ± 0.68 | c | Hydroxyl radical scavenging | 0.5 | SSPP-CE | 0.98 ± 1.39 | c |
| DPPH radical scavenging | 0.1 | SSPP-UW | 2.56 ± 0.90 | c | Hydroxyl radical scavenging | 0.5 | SSPP-UW | 0.49 ± 0.69 | c |
| DPPH radical scavenging | 0.1 | SSPP-UD | 3.45 ± 0.63 | c | Hydroxyl radical scavenging | 0.5 | SSPP-UD | 4.41 ± 0.64 | c |
| DPPH radical scavenging | 0.1 | BBP-CE | 2.98 ± 0.58 | c | Hydroxyl radical scavenging | 0.5 | BBP-CE | 9.78 ± 0.51 | b |
| DPPH radical scavenging | 0.1 | BBP-UW | 1.39 ± 0.56 | c | Hydroxyl radical scavenging | 0.5 | BBP-UW | 2.87 ± 0.32 | c |
| DPPH radical scavenging | 0.1 | BBP-UD | 6.96 ± 0.62 | b | Hydroxyl radical scavenging | 0.5 | BBP-UD | 5.80 ± 2.05 | bc |
| DPPH radical scavenging | 0.2 | Vc | 43.49 ± 1.93 | a | Hydroxyl radical scavenging | 1.0 | Vc | 63.37 ± 0.82 | a |
| DPPH radical scavenging | 0.2 | SSPP-CE | 4.14 ± 0.36 | c | Hydroxyl radical scavenging | 1.0 | SSPP-CE | 5.39 ± 0.69 | c |
| DPPH radical scavenging | 0.2 | SSPP-UW | 5.07 ± 1.01 | c | Hydroxyl radical scavenging | 1.0 | SSPP-UW | 4.41 ± 2.08 | c |
| DPPH radical scavenging | 0.2 | SSPP-UD | 7.97 ± 0.49 | b | Hydroxyl radical scavenging | 1.0 | SSPP-UD | 11.27 ± 0.64 | b |
| DPPH radical scavenging | 0.2 | BBP-CE | 7.22 ± 0.68 | b | Hydroxyl radical scavenging | 1.0 | BBP-CE | 12.32 ± 0.25 | b |
| DPPH radical scavenging | 0.2 | BBP-UW | 2.53 ± 0.70 | c | Hydroxyl radical scavenging | 1.0 | BBP-UW | 9.42 ± 0.73 | bc |
| DPPH radical scavenging | 0.2 | BBP-UD | 10.40 ± 0.89 | b | Hydroxyl radical scavenging | 1.0 | BBP-UD | 13.04 ± 1.02 | b |
| DPPH radical scavenging | 0.3 | Vc | 65.62 ± 0.52 | a | Hydroxyl radical scavenging | 1.5 | Vc | 70.35 ± 2.47 | a |
| DPPH radical scavenging | 0.3 | SSPP-CE | 6.29 ± 0.36 | d | Hydroxyl radical scavenging | 1.5 | SSPP-CE | 8.33 ± 2.05 | c |
| DPPH radical scavenging | 0.3 | SSPP-UW | 7.31 ± 1.30 | d | Hydroxyl radical scavenging | 1.5 | SSPP-UW | 11.76 ± 1.36 | c |
| DPPH radical scavenging | 0.3 | SSPP-UD | 11.82 ± 0.75 | c | Hydroxyl radical scavenging | 1.5 | SSPP-UD | 15.20 ± 2.08 | bc |
| DPPH radical scavenging | 0.3 | BBP-CE | 9.94 ± 1.02 | cd | Hydroxyl radical scavenging | 1.5 | BBP-CE | 14.13 ± 0.76 | bc |
| DPPH radical scavenging | 0.3 | BBP-UW | 4.63 ± 0.42 | d | Hydroxyl radical scavenging | 1.5 | BBP-UW | 17.39 ± 1.01 | bc |
| DPPH radical scavenging | 0.3 | BBP-UD | 15.08 ± 0.43 | b | Hydroxyl radical scavenging | 1.5 | BBP-UD | 18.84 ± 2.05 | b |
| DPPH radical scavenging | 0.4 | Vc | 87.64 ± 1.98 | a | Hydroxyl radical scavenging | 2.0 | Vc | 71.12 ± 0.82 | a |
| DPPH radical scavenging | 0.4 | SSPP-CE | 8.50 ± 0.37 | cd | Hydroxyl radical scavenging | 2.0 | SSPP-CE | 15.20 ± 2.08 | c |
| DPPH radical scavenging | 0.4 | SSPP-UW | 10.42 ± 1.27 | cd | Hydroxyl radical scavenging | 2.0 | SSPP-UW | 16.67 ± 1.36 | c |
| DPPH radical scavenging | 0.4 | SSPP-UD | 14.42 ± 0.91 | bc | Hydroxyl radical scavenging | 2.0 | SSPP-UD | 25.49 ± 1.39 | b |
| DPPH radical scavenging | 0.4 | BBP-CE | 12.21 ± 1.17 | c | Hydroxyl radical scavenging | 2.0 | BBP-CE | 14.86 ± 1.54 | c |
| DPPH radical scavenging | 0.4 | BBP-UW | 5.98 ± 0.36 | d | Hydroxyl radical scavenging | 2.0 | BBP-UW | 20.28 ± 1.42 | bc |
| DPPH radical scavenging | 0.4 | BBP-UD | 18.62 ± 1.32 | b | Hydroxyl radical scavenging | 2.0 | BBP-UD | 21.38 ± 0.76 | bc |
| DPPH radical scavenging | 0.5 | Vc | 99.92 ± 1.24 | a | Hydroxyl radical scavenging | 2.5 | Vc | 73.26 ± 0.55 | a |
| DPPH radical scavenging | 0.5 | SSPP-CE | 9.85 ± 1.10 | de | Hydroxyl radical scavenging | 2.5 | SSPP-CE | 20.59 ± 1.36 | cd |
| DPPH radical scavenging | 0.5 | SSPP-UW | 12.61 ± 0.56 | d | Hydroxyl radical scavenging | 2.5 | SSPP-UW | 21.57 ± 1.39 | cd |
| DPPH radical scavenging | 0.5 | SSPP-UD | 18.07 ± 0.88 | bc | Hydroxyl radical scavenging | 2.5 | SSPP-UD | 30.88 ± 2.05 | b |
| DPPH radical scavenging | 0.5 | BBP-CE | 16.44 ± 1.53 | c | Hydroxyl radical scavenging | 2.5 | BBP-CE | 17.03 ± 1.54 | d |
| DPPH radical scavenging | 0.5 | BBP-UW | 6.77 ± 0.36 | e | Hydroxyl radical scavenging | 2.5 | BBP-UW | 23.13 ± 1.33 | c |
| DPPH radical scavenging | 0.5 | BBP-UD | 20.35 ± 0.19 | b | Hydroxyl radical scavenging | 2.5 | BBP-UD | 24.28 ± 1.54 | c |
| DPPH radical scavenging | 1.0 | Vc | 99.91 ± 1.46 | a | Hydroxyl radical scavenging | 3.0 | Vc | 73.84 ± 0.27 | a |
| DPPH radical scavenging | 1.0 | SSPP-CE | 11.26 ± 1.82 | cd | Hydroxyl radical scavenging | 3.0 | SSPP-CE | 26.47 ± 1.39 | c |
| DPPH radical scavenging | 1.0 | SSPP-UW | 14.80 ± 1.54 | c | Hydroxyl radical scavenging | 3.0 | SSPP-UW | 25.00 ± 2.08 | cd |
| DPPH radical scavenging | 1.0 | SSPP-UD | 19.96 ± 0.27 | b | Hydroxyl radical scavenging | 3.0 | SSPP-UD | 33.33 ± 1.36 | b |
| DPPH radical scavenging | 1.0 | BBP-CE | 19.62 ± 1.00 | b | Hydroxyl radical scavenging | 3.0 | BBP-CE | 19.93 ± 1.54 | d |
| DPPH radical scavenging | 1.0 | BBP-UW | 7.44 ± 0.76 | d | Hydroxyl radical scavenging | 3.0 | BBP-UW | 26.09 ± 0.99 | c |
| DPPH radical scavenging | 1.0 | BBP-UD | 23.81 ± 0.43 | b | Hydroxyl radical scavenging | 3.0 | BBP-UD | 28.99 ± 2.05 | bc |
| DPPH radical scavenging | 1.5 | Vc | 99.91 ± 1.33 | a | Ferric reducing antioxidant power | 0.5 | Vc | 13.85 ± 0.51 | a |
| DPPH radical scavenging | 1.5 | SSPP-CE | 12.39 ± 0.37 | d | Ferric reducing antioxidant power | 0.5 | SSPP-CE | 0.10 ± 0.00 | c |
| DPPH radical scavenging | 1.5 | SSPP-UW | 18.00 ± 0.56 | c | Ferric reducing antioxidant power | 0.5 | SSPP-UW | 0.12 ± 0.02 | bc |
| DPPH radical scavenging | 1.5 | SSPP-UD | 21.54 ± 0.01 | bc | Ferric reducing antioxidant power | 0.5 | SSPP-UD | 0.89 ± 0.04 | b |
| DPPH radical scavenging | 1.5 | BBP-CE | 23.75 ± 1.54 | b | Ferric reducing antioxidant power | 0.5 | BBP-CE | 0.16 ± 0.01 | bc |
| DPPH radical scavenging | 1.5 | BBP-UW | 7.89 ± 0.07 | e | Ferric reducing antioxidant power | 0.5 | BBP-UW | 0.04 ± 0.02 | c |
| DPPH radical scavenging | 1.5 | BBP-UD | 24.43 ± 1.49 | b | Ferric reducing antioxidant power | 0.5 | BBP-UD | 0.75 ± 0.01 | bc |
| DPPH radical scavenging | 2.0 | Vc | 99.70 ± 0.91 | a | Ferric reducing antioxidant power | 1.0 | Vc | 23.86 ± 0.22 | a |
| DPPH radical scavenging | 2.0 | SSPP-CE | 13.25 ± 0.31 | d | Ferric reducing antioxidant power | 1.0 | SSPP-CE | 0.17 ± 0.01 | c |
| DPPH radical scavenging | 2.0 | SSPP-UW | 19.47 ± 2.53 | c | Ferric reducing antioxidant power | 1.0 | SSPP-UW | 0.22 ± 0.03 | c |
| DPPH radical scavenging | 2.0 | SSPP-UD | 22.46 ± 0.42 | bc | Ferric reducing antioxidant power | 1.0 | SSPP-UD | 1.73 ± 0.05 | b |
| DPPH radical scavenging | 2.0 | BBP-CE | 24.82 ± 0.87 | bc | Ferric reducing antioxidant power | 1.0 | BBP-CE | 0.32 ± 0.03 | c |
| DPPH radical scavenging | 2.0 | BBP-UW | 8.99 ± 0.05 | d | Ferric reducing antioxidant power | 1.0 | BBP-UW | 0.05 ± 0.00 | c |
| DPPH radical scavenging | 2.0 | BBP-UD | 25.56 ± 2.42 | b | Ferric reducing antioxidant power | 1.0 | BBP-UD | 1.51 ± 0.03 | b |
| DPPH radical scavenging | 2.5 | Vc | 99.79 ± 0.70 | a | Ferric reducing antioxidant power | 1.5 | Vc | 24.65 ± 0.07 | a |
| DPPH radical scavenging | 2.5 | SSPP-CE | 14.87 ± 1.33 | d | Ferric reducing antioxidant power | 1.5 | SSPP-CE | 0.25 ± 0.02 | ef |
| DPPH radical scavenging | 2.5 | SSPP-UW | 20.54 ± 1.07 | c | Ferric reducing antioxidant power | 1.5 | SSPP-UW | 0.32 ± 0.04 | e |
| DPPH radical scavenging | 2.5 | SSPP-UD | 24.26 ± 0.32 | bc | Ferric reducing antioxidant power | 1.5 | SSPP-UD | 2.77 ± 0.06 | b |
| DPPH radical scavenging | 2.5 | BBP-CE | 26.26 ± 2.06 | b | Ferric reducing antioxidant power | 1.5 | BBP-CE | 0.51 ± 0.02 | d |
| DPPH radical scavenging | 2.5 | BBP-UW | 10.60 ± 1.24 | d | Ferric reducing antioxidant power | 1.5 | BBP-UW | 0.08 ± 0.00 | f |
| DPPH radical scavenging | 2.5 | BBP-UD | 27.41 ± 1.12 | b | Ferric reducing antioxidant power | 1.5 | BBP-UD | 2.35 ± 0.04 | c |
| DPPH radical scavenging | 3.0 | Vc | 99.61 ± 1.89 | a | Ferric reducing antioxidant power | 2.0 | Vc | 24.65 ± 0.34 | a |
| DPPH radical scavenging | 3.0 | SSPP-CE | 16.52 ± 2.45 | c | Ferric reducing antioxidant power | 2.0 | SSPP-CE | 0.32 ± 0.01 | c |
| DPPH radical scavenging | 3.0 | SSPP-UW | 22.19 ± 1.43 | bc | Ferric reducing antioxidant power | 2.0 | SSPP-UW | 0.39 ± 0.04 | c |
| DPPH radical scavenging | 3.0 | SSPP-UD | 27.43 ± 0.29 | b | Ferric reducing antioxidant power | 2.0 | SSPP-UD | 3.30 ± 0.05 | b |
| DPPH radical scavenging | 3.0 | BBP-CE | 27.14 ± 1.14 | b | Ferric reducing antioxidant power | 2.0 | BBP-CE | 0.69 ± 0.00 | c |
| DPPH radical scavenging | 3.0 | BBP-UW | 11.11 ± 0.46 | c | Ferric reducing antioxidant power | 2.0 | BBP-UW | 0.09 ± 0.00 | c |
| DPPH radical scavenging | 3.0 | BBP-UD | 28.12 ± 2.25 | b | Ferric reducing antioxidant power | 2.0 | BBP-UD | 2.77 ± 0.10 | b |
| ABTS•⁺ scavenging | 0.1 | Vc | 22.31±1.07 | a | Ferric reducing antioxidant power | 2.5 | Vc | 24.63 ± 0.21 | a |
| ABTS•⁺ scavenging | 0.1 | SSPP-CE | 0.34±0.06 | b | Ferric reducing antioxidant power | 2.5 | SSPP-CE | 0.40 ± 0.02 | e |
| ABTS•⁺ scavenging | 0.1 | SSPP-UW | 0.23±0.06 | b | Ferric reducing antioxidant power | 2.5 | SSPP-UW | 0.55 ± 0.04 | de |
| ABTS•⁺ scavenging | 0.1 | SSPP-UD | 1.37±0.56 | b | Ferric reducing antioxidant power | 2.5 | SSPP-UD | 4.07 ± 0.07 | b |
| ABTS•⁺ scavenging | 0.1 | BBP-CE | 0.28±0.09 | b | Ferric reducing antioxidant power | 2.5 | BBP-CE | 0.92 ± 0.00 | d |
| ABTS•⁺ scavenging | 0.1 | BBP-UW | 0.93±0.09 | b | Ferric reducing antioxidant power | 2.5 | BBP-UW | 0.15 ± 0.00 | e |
| ABTS•⁺ scavenging | 0.1 | BBP-UD | 1.56±0.33 | b | Ferric reducing antioxidant power | 2.5 | BBP-UD | 3.47 ± 0.15 | c |
| ABTS•⁺ scavenging | 0.2 | Vc | 45.63±1.47 | a | Ferric reducing antioxidant power | 3.0 | Vc | 24.56 ± 0.41 | a |
| ABTS•⁺ scavenging | 0.2 | SSPP-CE | 0.62±0.09 | c | Ferric reducing antioxidant power | 3.0 | SSPP-CE | 0.44 ± 0.01 | de |
| ABTS•⁺ scavenging | 0.2 | SSPP-UW | 0.45±0.07 | c | Ferric reducing antioxidant power | 3.0 | SSPP-UW | 0.57 ± 0.04 | de |
| ABTS•⁺ scavenging | 0.2 | SSPP-UD | 3.77±0.72 | b | Ferric reducing antioxidant power | 3.0 | SSPP-UD | 4.80 ± 0.11 | b |
| ABTS•⁺ scavenging | 0.2 | BBP-CE | 0.45±0.07 | c | Ferric reducing antioxidant power | 3.0 | BBP-CE | 1.03 ± 0.02 | d |
| ABTS•⁺ scavenging | 0.2 | BBP-UW | 1.88±0.21 | bc | Ferric reducing antioxidant power | 3.0 | BBP-UW | 0.15 ± 0.01 | e |
| ABTS•⁺ scavenging | 0.2 | BBP-UD | 2.55±0.62 | bc | Ferric reducing antioxidant power | 3.0 | BBP-UD | 4.09 ± 0.11 | c |
| ABTS•⁺ scavenging | 0.3 | Vc | 69.59±0.91 | a |  |  |  |  |  |
| ABTS•⁺ scavenging | 0.3 | SSPP-CE | 0.89±0.06 | d |  |  |  |  |  |
| ABTS•⁺ scavenging | 0.3 | SSPP-UW | 0.70±0.11 | d |  |  |  |  |  |
| ABTS•⁺ scavenging | 0.3 | SSPP-UD | 5.55±0.48 | b |  |  |  |  |  |
| ABTS•⁺ scavenging | 0.3 | BBP-CE | 0.70±0.10 | d |  |  |  |  |  |
| ABTS•⁺ scavenging | 0.3 | BBP-UW | 2.87±0.38 | c |  |  |  |  |  |
| ABTS•⁺ scavenging | 0.3 | BBP-UD | 3.55±0.48 | c |  |  |  |  |  |
| ABTS•⁺ scavenging | 0.4 | Vc | 88.31±1.49 | a |  |  |  |  |  |
| ABTS•⁺ scavenging | 0.4 | SSPP-CE | 1.42±0.21 | c |  |  |  |  |  |
| ABTS•⁺ scavenging | 0.4 | SSPP-UW | 0.90±0.14 | c |  |  |  |  |  |
| ABTS•⁺ scavenging | 0.4 | SSPP-UD | 7.34±0.84 | b |  |  |  |  |  |
| ABTS•⁺ scavenging | 0.4 | BBP-CE | 0.89±0.12 | c |  |  |  |  |  |
| ABTS•⁺ scavenging | 0.4 | BBP-UW | 3.86±0.76 | c |  |  |  |  |  |
| ABTS•⁺ scavenging | 0.4 | BBP-UD | 4.75±0.73 | bc |  |  |  |  |  |
| ABTS•⁺ scavenging | 0.5 | Vc | 99.57±0.35 | a |  |  |  |  |  |
| ABTS•⁺ scavenging | 0.5 | SSPP-CE | 1.84±0.09 | cd |  |  |  |  |  |
| ABTS•⁺ scavenging | 0.5 | SSPP-UW | 1.24±0.07 | cd |  |  |  |  |  |
| ABTS•⁺ scavenging | 0.5 | SSPP-UD | 8.85±0.99 | b |  |  |  |  |  |
| ABTS•⁺ scavenging | 0.5 | BBP-CE | 1.13±0.08 | d |  |  |  |  |  |
| ABTS•⁺ scavenging | 0.5 | BBP-UW | 4.16±0.48 | c |  |  |  |  |  |
| ABTS•⁺ scavenging | 0.5 | BBP-UD | 6.02±1.58 | b |  |  |  |  |  |
| ABTS•⁺ scavenging | 1.0 | Vc | 99.40±2.04 | a |  |  |  |  |  |
| ABTS•⁺ scavenging | 1.0 | SSPP-CE | 3.90±0.68 | c |  |  |  |  |  |
| ABTS•⁺ scavenging | 1.0 | SSPP-UW | 4.00±0.66 | c |  |  |  |  |  |
| ABTS•⁺ scavenging | 1.0 | SSPP-UD | 11.39±1.59 | b |  |  |  |  |  |
| ABTS•⁺ scavenging | 1.0 | BBP-CE | 2.85±0.67 | c |  |  |  |  |  |
| ABTS•⁺ scavenging | 1.0 | BBP-UW | 4.82±0.01 | c |  |  |  |  |  |
| ABTS•⁺ scavenging | 1.0 | BBP-UD | 8.87±2.31 | bc |  |  |  |  |  |
| ABTS•⁺ scavenging | 1.5 | Vc | 99.49±1.79 | a |  |  |  |  |  |
| ABTS•⁺ scavenging | 1.5 | SSPP-CE | 6.13±0.38 | c |  |  |  |  |  |
| ABTS•⁺ scavenging | 1.5 | SSPP-UW | 5.52±1.27 | c |  |  |  |  |  |
| ABTS•⁺ scavenging | 1.5 | SSPP-UD | 12.58±0.41 | b |  |  |  |  |  |
| ABTS•⁺ scavenging | 1.5 | BBP-CE | 4.95±0.33 | c |  |  |  |  |  |
| ABTS•⁺ scavenging | 1.5 | BBP-UW | 6.98±0.60 | c |  |  |  |  |  |
| ABTS•⁺ scavenging | 1.5 | BBP-UD | 10.87±0.89 | b |  |  |  |  |  |
| ABTS•⁺ scavenging | 2.0 | Vc | 99.50±1.04 | a |  |  |  |  |  |
| ABTS•⁺ scavenging | 2.0 | SSPP-CE | 11.26±0.20 | bc |  |  |  |  |  |
| ABTS•⁺ scavenging | 2.0 | SSPP-UW | 7.50±1.12 | c |  |  |  |  |  |
| ABTS•⁺ scavenging | 2.0 | SSPP-UD | 14.41±1.25 | b |  |  |  |  |  |
| ABTS•⁺ scavenging | 2.0 | BBP-CE | 8.23±1.38 | c |  |  |  |  |  |
| ABTS•⁺ scavenging | 2.0 | BBP-UW | 7.93±0.06 | c |  |  |  |  |  |
| ABTS•⁺ scavenging | 2.0 | BBP-UD | 12.55±0.20 | b |  |  |  |  |  |
| ABTS•⁺ scavenging | 2.5 | Vc | 99.77±1.09 | a |  |  |  |  |  |
| ABTS•⁺ scavenging | 2.5 | SSPP-CE | 12.06±1.37 | c |  |  |  |  |  |
| ABTS•⁺ scavenging | 2.5 | SSPP-UW | 13.59±2.15 | c |  |  |  |  |  |
| ABTS•⁺ scavenging | 2.5 | SSPP-UD | 17.01±2.08 | b |  |  |  |  |  |
| ABTS•⁺ scavenging | 2.5 | BBP-CE | 13.85±2.06 | c |  |  |  |  |  |
| ABTS•⁺ scavenging | 2.5 | BBP-UW | 8.58±1.31 | c |  |  |  |  |  |
| ABTS•⁺ scavenging | 2.5 | BBP-UD | 14.86±0.40 | b |  |  |  |  |  |
| ABTS•⁺ scavenging | 3.0 | Vc | 99.80±1.53 | a |  |  |  |  |  |
| ABTS•⁺ scavenging | 3.0 | SSPP-CE | 12.20±0.72 | c |  |  |  |  |  |
| ABTS•⁺ scavenging | 3.0 | SSPP-UW | 17.31±2.20 | bc |  |  |  |  |  |
| ABTS•⁺ scavenging | 3.0 | SSPP-UD | 20.62±2.14 | b |  |  |  |  |  |
| ABTS•⁺ scavenging | 3.0 | BBP-CE | 16.81±2.10 | bc |  |  |  |  |  |
| ABTS•⁺ scavenging | 3.0 | BBP-UW | 9.42±0.10 | c |  |  |  |  |  |
| ABTS•⁺ scavenging | 3.0 | BBP-UD | 18.02±1.26 | bc |  |  |  |  |  |

*Values are expressed as mean ± SD (n = 3). Statistical analysis was performed using one-way ANOVA followed by Tukey’s multiple comparison test. Different lowercase letters indicate significant differences among samples at the same concentration within each assay (*p* < 0.05).

**Table S6. Concentration-dependent inhibitory activities of different samples against *α*-amylase and *α*-glucosidase with statistical comparisons***.

| Assay | Concentration | Sample | Inhibition rate (%) | Group | Assay | Concentration | Sample | Inhibition rate (%) | Group |
| --- | --- | --- | --- | --- | --- | --- | --- | --- | --- |
| *α*-amylase inhibitory | 0.25 | Acarbose | 53.23 ± 0.66 | a | *α*-glucosidase inhibitory | 0.25 | Acarbose | 74.53 ± 1.76 | a |
| *α*-amylase inhibitory | 0.25 | SSPP-CE | 0.74 ± 0.07 | d | *α*-glucosidase inhibitory | 0.25 | SSPP-CE | 1.58 ± 0.45 | d |
| *α*-amylase inhibitory | 0.25 | SSPP-UW | 2.77 ± 0.28 | d | *α*-glucosidase inhibitory | 0.25 | SSPP-UW | 31.17 ± 0.99 | b |
| *α*-amylase inhibitory | 0.25 | SSPP-UD | 25.15 ± 0.78 | b | *α*-glucosidase inhibitory | 0.25 | SSPP-UD | 28.07 ± 1.18 | b |
| *α*-amylase inhibitory | 0.25 | BBP-CE | 0.19 ± 0.07 | d | *α*-glucosidase inhibitory | 0.25 | BBP-CE | 4.38 ± 0.55 | d |
| *α*-amylase inhibitory | 0.25 | BBP-UW | 0.73 ± 0.08 | d | *α*-glucosidase inhibitory | 0.25 | BBP-UW | 4.80 ± 0.78 | d |
| *α*-amylase inhibitory | 0.25 | BBP-UD | 6.43 ± 1.89 | c | *α*-glucosidase inhibitory | 0.25 | BBP-UD | 13.06 ± 0.86 | c |
| *α*-amylase inhibitory | 0.5 | Acarbose | 64.34 ± 0.52 | a | *α*-glucosidase inhibitory | 0.50 | Acarbose | 82.13 ± 0.50 | a |
| *α*-amylase inhibitory | 0.5 | SSPP-CE | 5.88 ± 0.96 | d | *α*-glucosidase inhibitory | 0.50 | SSPP-CE | 5.40 ± 0.71 | e |
| *α*-amylase inhibitory | 0.5 | SSPP-UW | 10.29 ± 2.08 | d | *α*-glucosidase inhibitory | 0.50 | SSPP-UW | 43.92 ± 1.36 | b |
| *α*-amylase inhibitory | 0.5 | SSPP-UD | 39.46 ± 1.23 | b | *α*-glucosidase inhibitory | 0.50 | SSPP-UD | 43.59 ± 1.16 | b |
| *α*-amylase inhibitory | 0.5 | BBP-CE | 4.78 ± 2.60 | d | *α*-glucosidase inhibitory | 0.50 | BBP-CE | 9.58 ± 0.66 | d |
| *α*-amylase inhibitory | 0.5 | BBP-UW | 4.41 ± 0.08 | d | *α*-glucosidase inhibitory | 0.50 | BBP-UW | 11.98 ± 1.59 | d |
| *α*-amylase inhibitory | 0.5 | BBP-UD | 17.65 ± 1.71 | c | *α*-glucosidase inhibitory | 0.50 | BBP-UD | 20.14 ± 0.50 | c |
| *α*-amylase inhibitory | 1.0 | Acarbose | 73.69 ± 1.85 | a | *α*-glucosidase inhibitory | 1.00 | Acarbose | 85.98 ± 2.55 | a |
| *α*-amylase inhibitory | 1.0 | SSPP-CE | 16.18 ± 2.08 | d | *α*-glucosidase inhibitory | 1.00 | SSPP-CE | 10.65 ± 1.06 | e |
| *α*-amylase inhibitory | 1.0 | SSPP-UW | 20.96 ± 1.85 | d | *α*-glucosidase inhibitory | 1.00 | SSPP-UW | 56.83 ± 2.39 | b |
| *α*-amylase inhibitory | 1.0 | SSPP-UD | 54.41 ± 0.08 | b | *α*-glucosidase inhibitory | 1.00 | SSPP-UD | 57.69 ± 1.59 | b |
| *α*-amylase inhibitory | 1.0 | BBP-CE | 10.66 ± 0.44 | d | *α*-glucosidase inhibitory | 1.00 | BBP-CE | 19.44 ± 0.66 | d |
| *α*-amylase inhibitory | 1.0 | BBP-UW | 7.72 ± 2.60 | d | *α*-glucosidase inhibitory | 1.00 | BBP-UW | 26.70 ± 1.53 | c |
| *α*-amylase inhibitory | 1.0 | BBP-UD | 27.57 ± 2.60 | c | *α*-glucosidase inhibitory | 1.00 | BBP-UD | 28.94 ± 1.89 | c |
| *α*-amylase inhibitory | 1.5 | Acarbose | 77.57 ± 0.81 | a | *α*-glucosidase inhibitory | 1.50 | Acarbose | 86.54 ± 1.35 | a |
| *α*-amylase inhibitory | 1.5 | SSPP-CE | 23.53 ± 1.71 | d | *α*-glucosidase inhibitory | 1.50 | SSPP-CE | 17.66 ± 0.50 | e |
| *α*-amylase inhibitory | 1.5 | SSPP-UW | 31.62 ± 2.08 | c | *α*-glucosidase inhibitory | 1.50 | SSPP-UW | 59.93 ± 2.57 | b |
| *α*-amylase inhibitory | 1.5 | SSPP-UD | 61.40 ± 0.52 | b | *α*-glucosidase inhibitory | 1.50 | SSPP-UD | 66.27 ± 1.99 | b |
| *α*-amylase inhibitory | 1.5 | BBP-CE | 18.01 ± 2.23 | de | *α*-glucosidase inhibitory | 1.50 | BBP-CE | 27.89 ± 1.49 | d |
| *α*-amylase inhibitory | 1.5 | BBP-UW | 15.07 ± 0.52 | e | *α*-glucosidase inhibitory | 1.50 | BBP-UW | 33.57 ± 0.20 | cd |
| *α*-amylase inhibitory | 1.5 | BBP-UD | 35.66 ± 2.23 | c | *α*-glucosidase inhibitory | 1.50 | BBP-UD | 35.74 ± 2.64 | c |
| *α*-amylase inhibitory | 2.0 | Acarbose | 81.62 ± 1.71 | a | *α*-glucosidase inhibitory | 2.00 | Acarbose | 91.94 ± 2.48 | a |
| *α*-amylase inhibitory | 2.0 | SSPP-CE | 32.35 ± 1.33 | d | *α*-glucosidase inhibitory | 2.00 | SSPP-CE | 23.97 ± 1.49 | e |
| *α*-amylase inhibitory | 2.0 | SSPP-UW | 45.96 ± 0.52 | c | *α*-glucosidase inhibitory | 2.00 | SSPP-UW | 64.51 ± 2.49 | b |
| *α*-amylase inhibitory | 2.0 | SSPP-UD | 72.06 ± 0.96 | b | *α*-glucosidase inhibitory | 2.00 | SSPP-UD | 70.14 ± 0.24 | b |
| *α*-amylase inhibitory | 2.0 | BBP-CE | 27.21 ± 1.04 | de | *α*-glucosidase inhibitory | 2.00 | BBP-CE | 34.58 ± 1.99 | d |
| *α*-amylase inhibitory | 2.0 | BBP-UW | 23.90 ± 2.23 | e | *α*-glucosidase inhibitory | 2.00 | BBP-UW | 44.22 ± 0.54 | c |
| *α*-amylase inhibitory | 2.0 | BBP-UD | 46.69 ± 2.60 | c | *α*-glucosidase inhibitory | 2.00 | BBP-UD | 42.68 ± 0.50 | c |
| *α*-amylase inhibitory | 2.5 | Acarbose | 88.60 ± 2.23 | a | *α*-glucosidase inhibitory | 2.50 | Acarbose | 93.74 ± 1.21 | a |
| *α*-amylase inhibitory | 2.5 | SSPP-CE | 41.91 ± 1.71 | d | *α*-glucosidase inhibitory | 2.50 | SSPP-CE | 26.77 ± 1.49 | e |
| *α*-amylase inhibitory | 2.5 | SSPP-UW | 53.68 ± 0.08 | c | *α*-glucosidase inhibitory | 2.50 | SSPP-UW | 70.49 ± 1.00 | c |
| *α*-amylase inhibitory | 2.5 | SSPP-UD | 80.51 ± 1.56 | b | *α*-glucosidase inhibitory | 2.50 | SSPP-UD | 78.24 ± 1.99 | b |
| *α*-amylase inhibitory | 2.5 | BBP-CE | 39.34 ± 1.85 | d | *α*-glucosidase inhibitory | 2.50 | BBP-CE | 43.73 ± 2.57 | d |
| *α*-amylase inhibitory | 2.5 | BBP-UW | 37.87 ± 2.60 | d | *α*-glucosidase inhibitory | 2.50 | BBP-UW | 49.23 ± 2.53 | d |
| *α*-amylase inhibitory | 2.5 | BBP-UD | 54.04 ± 0.81 | c | *α*-glucosidase inhibitory | 2.50 | BBP-UD | 50.42 ± 0.18 | d |
| *α*-amylase inhibitory | 3.0 | Acarbose | 93.01 ± 2.23 | a | *α*-glucosidase inhibitory | 3.00 | Acarbose | 95.80 ± 0.14 | a |
| *α*-amylase inhibitory | 3.0 | SSPP-CE | 50.00 ± 2.08 | c | *α*-glucosidase inhibitory | 3.00 | SSPP-CE | 37.98 ± 1.63 | e |
| *α*-amylase inhibitory | 3.0 | SSPP-UW | 65.44 ± 0.96 | b | *α*-glucosidase inhibitory | 3.00 | SSPP-UW | 77.32 ± 0.70 | c |
| *α*-amylase inhibitory | 3.0 | SSPP-UD | 87.13 ± 2.60 | a | *α*-glucosidase inhibitory | 3.00 | SSPP-UD | 84.93 ± 0.50 | b |
| *α*-amylase inhibitory | 3.0 | BBP-CE | 52.21 ± 1.71 | c | *α*-glucosidase inhibitory | 3.00 | BBP-CE | 50.77 ± 1.00 | d |
| *α*-amylase inhibitory | 3.0 | BBP-UW | 48.90 ± 2.23 | c | *α*-glucosidase inhibitory | 3.00 | BBP-UW | 58.86 ± 0.79 | d |
| *α*-amylase inhibitory | 3.0 | BBP-UD | 63.24 ± 2.08 | b | *α*-glucosidase inhibitory | 3.00 | BBP-UD | 62.75 ± 1.99 | d |
| *α*-amylase inhibitory | 3.5 | Acarbose | 98.66 ± 1.35 | a | *α*-glucosidase inhibitory | 3.50 | Acarbose | 99.19 ± 0.71 | a |
| *α*-amylase inhibitory | 3.5 | SSPP-CE | 63.89 ± 1.42 | c | *α*-glucosidase inhibitory | 3.50 | SSPP-CE | 49.24 ± 0.63 | g |
| *α*-amylase inhibitory | 3.5 | SSPP-UW | 77.84 ± 1.20 | b | *α*-glucosidase inhibitory | 3.50 | SSPP-UW | 86.19 ± 0.71 | c |
| *α*-amylase inhibitory | 3.5 | SSPP-UD | 96.75 ± 1.47 | a | *α*-glucosidase inhibitory | 3.50 | SSPP-UD | 93.73 ± 1.32 | b |
| *α*-amylase inhibitory | 3.5 | BBP-CE | 68.29 ± 0.81 | c | *α*-glucosidase inhibitory | 3.50 | BBP-CE | 59.84 ± 1.63 | f |
| *α*-amylase inhibitory | 3.5 | BBP-UW | 58.68 ± 1.40 | d | *α*-glucosidase inhibitory | 3.50 | BBP-UW | 65.74 ± 1.34 | e |
| *α*-amylase inhibitory | 3.5 | BBP-UD | 77.68 ± 1.41 | b | *α*-glucosidase inhibitory | 3.50 | BBP-UD | 72.18 ± 2.13 | d |

*Values are expressed as mean ± SD (n = 3). Statistical analysis was performed using one-way ANOVA followed by Tukey’s multiple comparison test. Different lowercase letters indicate significant differences among samples at the same concentration within each assay (*p* < 0.05).

**Table S7.** IC_50_ values of SSPP and BBP fractions for *α*‑amylase and *α*‑glucosidase inhibition, with 95% confidence intervals and R^2^ of the dose-response fits.

| Assay | Sample | IC_50_ (mg/mL) | 95% CI | R^2^ | Note |
| --- | --- | --- | --- | --- | --- |
| *α*-amylase inhibitory | Acarbose | 0.26 | 1.20-0.33 | 0.9224 | Determined |
| *α*-amylase inhibitory | SSPP-CE | 2.86 | 2.72-3.02 | 0.9823 | Determined |
| *α*-amylase inhibitory | SSPP-UW | 2.13 | 2.02-2.26 | 0.9802 | Determined |
| *α*-amylase inhibitory | SSPP-UD | 0.77 | 0.64-0.90 | 0.9416 | Determined |
| *α*-amylase inhibitory | BBP-CE | 2.82 | 2.71-2.95 | 0.9799 | Determined |
| *α*-amylase inhibitory | BBP-UW | 3.07 | 2.98-3.17 | 0.9905 | Determined |
| *α*-amylase inhibitory | BBP-UD | 2.02 | 1.84-2.21 | 0.9579 | Determined |
| *α*-glucosidase inhibitory | Acarbose | ND | — | — | Not reliably fitted |
| *α*-glucosidase inhibitory | SSPP-CE | >3.5 | — | — | Not reached |
| *α*-glucosidase inhibitory | SSPP-UW | 0.73 | 0.61-0.84 | 0.9401 | Determined |
| *α*-glucosidase inhibitory | SSPP-UD | 0.68 | 0.59-0.78 | 0.9552 | Determined |
| *α*-glucosidase inhibitory | BBP-CE | 2.90 | 2.74-3.08 | 0.9832 | Determined |
| *α*-glucosidase inhibitory | BBP-UW | 2.34 | 2.24-2.45 | 0.9897 | Determined |
| *α*-glucosidase inhibitory | BBP-UD | 2.13 | 1.89-2.41 | 0.9319 | Determined |

**References**

Becke, & Axel, D. (1993). Density‐functional thermochemistry. III. The role of exact exchange. *Journal of Chemical Physics, 98*(7), 5648-5652.

Chen, F., & Huang, G. (2019). Extraction, derivatization and antioxidant activity of bitter gourd polysaccharide. *Int J Biol Macromol, 141*, 14-20. <https://doi.org/10.1016/j.ijbiomac.2019.08.239>.

Frisch, M. J., Trucks, G. W., Schlegel, J., Scuseria, G. E., Robb, M. A., Cheeseman, J. R., . . . Mennucci, B. (2010). Gaussian 09, Revision C.01.

Geng, X., Guo, D., Wu, B., Wang, W., Zhang, D., Hou, S., . . . Chang, M. (2024). Effects of different extraction methods on the physico-chemical characteristics and biological activities of polysaccharides from Clitocybe squamulosa. *Int J Biol Macromol, 259*(Pt 2), 129234. <https://doi.org/10.1016/j.ijbiomac.2024.129234>.

González-Centeno, M. R., Jourdes, M., Femenia, A., Simal, S., Rosselló, C., & Teissedre, P. L. (2012). Proanthocyanidin composition and antioxidant potential of the stem winemaking byproducts from 10 different grape varieties (Vitis vinifera L.). *J Agric Food Chem, 60*(48), 11850-11858. <https://doi.org/10.1021/jf303047k>.

Humphrey, W., Dalke, A., & Schulten, K. (1996). VMD: Visual molecular dynamics. *j mol graph, 14*(1), 33-38.

Lee, C., Yang, W., & Parr, R. G. (1988). Development of the Colle-Salvetti correlation-energy formula into a functional of the electron density. *37*(2), 785-789.

Lu, T., & Chen, F. (2012). Multiwfn: A multifunctional wavefunction analyzer. *Journal of Computational Chemistry, 33*(5), 580-592.

Miehlich, B., Savin, A., Stoll, H., & Preuss, H. (1989). Results obtained with the correlation energy density functionals of becke and Lee, Yang and Parr. *Chemical Physics Letters, 157*(3), 200-206.

Mohammed, J. K., Mahdi, A. A., Ahmed, M. I., Ma, M., & Wang, H. (2020). Preparation, deproteinization, characterization, and antioxidant activity of polysaccharide from Medemia argun fruit. *Int J Biol Macromol, 155*, 919-926. <https://doi.org/10.1016/j.ijbiomac.2019.11.050>.

Saravanakumar, K., Park, S., Sathiyaseelan, A., Mariadoss, A. V. A., Park, S., Kim, S. J., & Wang, M. H. (2021). Isolation of Polysaccharides from Trichoderma harzianum with Antioxidant, Anticancer, and Enzyme Inhibition Properties. *Antioxidants (Basel), 10*(9). <https://doi.org/10.3390/antiox10091372>.

Schafer, A., Horn, H., & Ahlrichs, R. (1992). Fully optimized contracted Gaussian basis sets for atoms Li to Kr. *Journal of Chemical Physics, 97*, 2571-2577.

Wang, C., Santhanam, R. K., Gao, X., Chen, Z., Chen, Y., Wang, C., . . . Chen, H. (2018a). Preparation, characterization of polysaccharides fractions from Inonotus obliquus and their effects on α-amylase, α-glucosidase activity and H2O2-induced oxidative damage in hepatic L02 cells. *Journal of Functional Foods, 48*, 179-189.

Wang, L., Zhang, B., Xiao, J., Huang, Q., Li, C., & Fu, X. (2018b). Physicochemical, functional, and biological properties of water-soluble polysaccharides from Rosa roxburghii Tratt fruit. *Food Chem, 249*, 127-135. <https://doi.org/10.1016/j.foodchem.2018.01.011>.

Wang, Z. B., Pei, J. J., Ma, H. L., Cai, P. F., & Yan, J. K. (2014). Effect of extraction media on preliminary characterizations and antioxidant activities of Phellinus linteus polysaccharides. *Carbohydr Polym, 109*, 49-55. <https://doi.org/10.1016/j.carbpol.2014.03.057>.

Weigend, F., & Ahlrichs, R. (2005). Balanced basis sets of split valence, triple zeta valence and quadruple zeta valence quality for H to Rn: Design and assessment of accuracy. *Physical Chemistry Chemical Physics Pccp, 7*(18), 3297-3305.

Zhang, F., Zhang, L., Chen, J., Du, X., Lu, Z., Wang, X., . . . Lü, X. (2022). Systematic evaluation of a series of pectic polysaccharides extracted from apple pomace by regulation of subcritical water conditions. *Food Chem, 368*, 130833. <https://doi.org/10.1016/j.foodchem.2021.130833>.
